# In Situ Genetic Engineering Facilitates MMP‐9‐Responsive TIMP1 Release Through Proteolytic Cleavage to Boost Diabetic Wound Healing

**DOI:** 10.1002/advs.202524328

**Published:** 2026-09-27

**Authors:** Yuan Zhang, Jiani Deng, Man Yee Cheung, Tingting Fu, Xufeng Qi, Dongqing Cai, Jianmin Sun, Gang Lu, Peng Shi, Wai‐Yee Chan, Xu Li, Hui Zhao

**Affiliations:** ^1^ School of Biomedical Sciences Faculty of Medicine Key Laboratory for Regenerative Medicine, Ministry of Education of China The Chinese University of Hong Kong Hong Kong People's Republic of China; ^2^ Beijing Jishuitan Hospital Capital Medical University Beijing People's Republic of China; ^3^ National Infrastructures for Translational Medicine (Peking Union), Peking Union Medical College Hospital Chinese Academy of Medical Sciences & Peking Union Medical College Beijing People's Republic of China; ^4^ Department of Developmental & Regenerative Biology Key Laboratory of Regenerative Medicine, Ministry of Education Jinan University Guangzhou People's Republic of China; ^5^ Department of Pathogen Biology and Immunology, School of Basic Medical Sciences Ningxia Medical University Yinchuan People's Republic of China; ^6^ Kunming Institute of Zoology The Chinese University of Hong Kong Kunming Institute of Zoology ‐ The Chinese University of Hong Kong (KIZ‐CUHK) Joint Laboratory of Bioresources and Molecular Research of Common Diseases Hong Kong People's Republic of China; ^7^ Hong Kong Branch of CAS Center for Excellence in Animal Evolution and Genetics The Chinese University of Hong Kong Hong Kong People's Republic of China; ^8^ State Key Laboratory of Genetic Resources and Evolution/Yunnan Key Laboratory of Biodiversity Information Kunming Institute of Zoology The Chinese Academy of Sciences Kunming People's Republic of China

**Keywords:** diabetic foot ulcer, matrix metalloproteinase‐9, protein release system, tissue inhibitor of metalloproteinase 1, wound healing

## Abstract

Diabetic Foot Ulcer (DFU), a severe chronic diabetes complication with low healing and high recurrence rates, is a major global health challenge. Overexpression of matrix metalloproteinase‐9 (MMP‐9) and the consequent MMP‐9/TIMP1 (tissue inhibitor of metalloproteinase 1) imbalance delays healing by degrading the extracellular matrix, impairing granulation tissue formation, and exacerbating inflammation. Conventional therapies do not dynamically respond to fluctuating protease levels in chronic wounds. This study introduces an MMP‐9‐responsive protein release system (M9RR), where the therapeutic protein (i.e., TIMP1) is linked to a membrane‐anchoring domain at its C‐terminus via an MMP‐9‐cleavable peptide, thereby exposing the fusion protein to the extracellular side of cell membranes. The system enables targeted release of TIMP1 in high‐MMP‐9 microenvironments, thereby neutralizing excessive MMP‐9 activity. In vitro, M9RR demonstrates MMP‐9 specificity, broad mammalian cell applicability, and protection of HaCaT keratinocytes and BJ fibroblasts from MMP‐9‐induced damage. In db/db diabetic mice, the MMP‐9‐responsive TIMP1 release system (TIMP1^M9RR^) significantly improves wound contraction, granulation tissue formation, epithelial regeneration, and collagen remodeling. Additionally, a cryomicroneedle (CryoMNs)‐based co‐delivery system for basic fibroblast growth factor (bFGF) and TIMP1^M9RR^ shows effective diabetic wound healing. This modular M9RR system offers a precise, adaptive therapeutic strategy for DFU and holds promise for other MMP‐related diseases.

## Graphical abstract

This study develops an MMP‐9‐responsive protein release system that transforms resident cells into drug depots through in situ genetic engineering. Anchored to the membrane, the therapeutic protein TIMP1 is selectively released upon MMP‐9 cleavage. The system enhanced wound healing in diabetic mice and acted synergistically with bFGF upon cryomicroneedle delivery, offering a precise strategy for DFU. (By Figdraw)

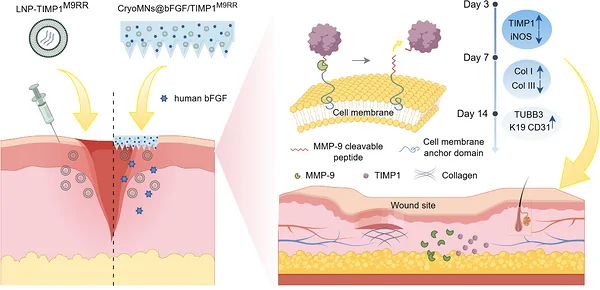

## 1 Introduction

Diabetic foot ulcer (DFU), a severe chronic complication of diabetes mellitus, presents a significant global public health challenge, with an incidence ranging from 19% to 34% among affected individuals [1]. Despite the implementation of standardized clinical treatment regimens, the wound healing rate of DFU patients remains less than 50%, and a recurrence rate can be as high as 40%, imposing a heavy burden on patients' physical and mental health as well as healthcare systems worldwide [2, 3].

Aberrant high levels of matrix metalloproteinase‐9 (MMP‐9, gelatinase B) in wound tissue have been recognized as a key factor in impaired healing of diabetic foot ulcers (DFUs) [4, 5, 6]. In addition, tissue inhibitor of metalloproteinase 1 (TIMP1), the endogenous inhibitor of MMP‐9, has also been found to be downregulated in diabetic wound tissue and in the serum of diabetic patients [5, 7]. This imbalance, reflected by an increased MMP‐9/TIMP‐1 ratio, is closely linked to impaired wound healing [8, 9]. Previous studies have shown that excessive MMP‐9 expression impairs granulation tissue formation, the primary connective tissue that fills wound defects during the early healing phase. Impaired granulation tissue, in turn, delays the initiation of wound repair [10]. Additionally, MMP‐9 has been reported to selectively inactivate key reparative growth factors, including vascular endothelial growth factor (VEGF) [10], and exacerbate local inflammation by promoting endothelial cell ferroptosis [11] and macrophage M1 polarization [9]. Notably, selective inhibition of MMP‐9 has been shown to accelerate wound healing in diabetic mouse models, further supporting its potential as a therapeutic target for DFU [12].

Current regulatory strategies targeting MMP‐9 in diabetic wounds primarily include modulating its proteolytic activity using chemical or natural inhibitors, as well as suppressing its transcription via siRNA delivery [12, 13, 14, 15, 16]. However, broad‐spectrum MMP inhibitors lack substrate specificity and are associated with adverse effects such as joint stiffness [16, 17]. Meanwhile, locally administered MMP‐9‐specific inhibitors cannot dynamically adjust dosage in response to fluctuating MMP‐9 levels during healing, and siRNA‐based strategies are similarly constrained by this unresponsive nature. Therefore, developing a responsive system that can detect real‐time changes in MMP‐9 and enable precise, on‐demand drug release is critical to advancing diabetic wound therapy.

In this study, we developed an MMP‐9‐responsive protein release system (M9RR). The therapeutic protein was conjugated to a transmembrane anchor domain via an MMP‐9‐cleavable peptide, thereby forming a membrane‐bound protein. Active MMP‐9 in the extracellular microenvironment cleaves the peptide linker, thereby releasing the therapeutic protein from the cell membrane and into the extracellular matrix. This system enables precise delivery of therapeutic protein (e.g., TIMP1) in the high‐MMP‐9 microenvironment of wounds. Compared to direct delivery of TIMP1 mRNA, the MMP‐9‐responsive TIMP1 release system (TIMP1^M9RR^) demonstrated superior pro‐healing efficacy in diabetic wound healing mouse models of diabetic wound healing. Building on this, a co‐delivery strategy was developed using cryomicroneedles (CryoMNs) to administer both TIMP1^M9RR^ and basic fibroblast growth factor (bFGF). In vivo application demonstrated that TIMP1^M9RR^ enhanced the therapeutic effect of bFGF, particularly in terms of inflammatory regulation and collagen remodeling.

## 2 Results

### 2.1 High MMP‐9 Expression Impedes Diabetic Wound Healing

**FIGURE 1:**
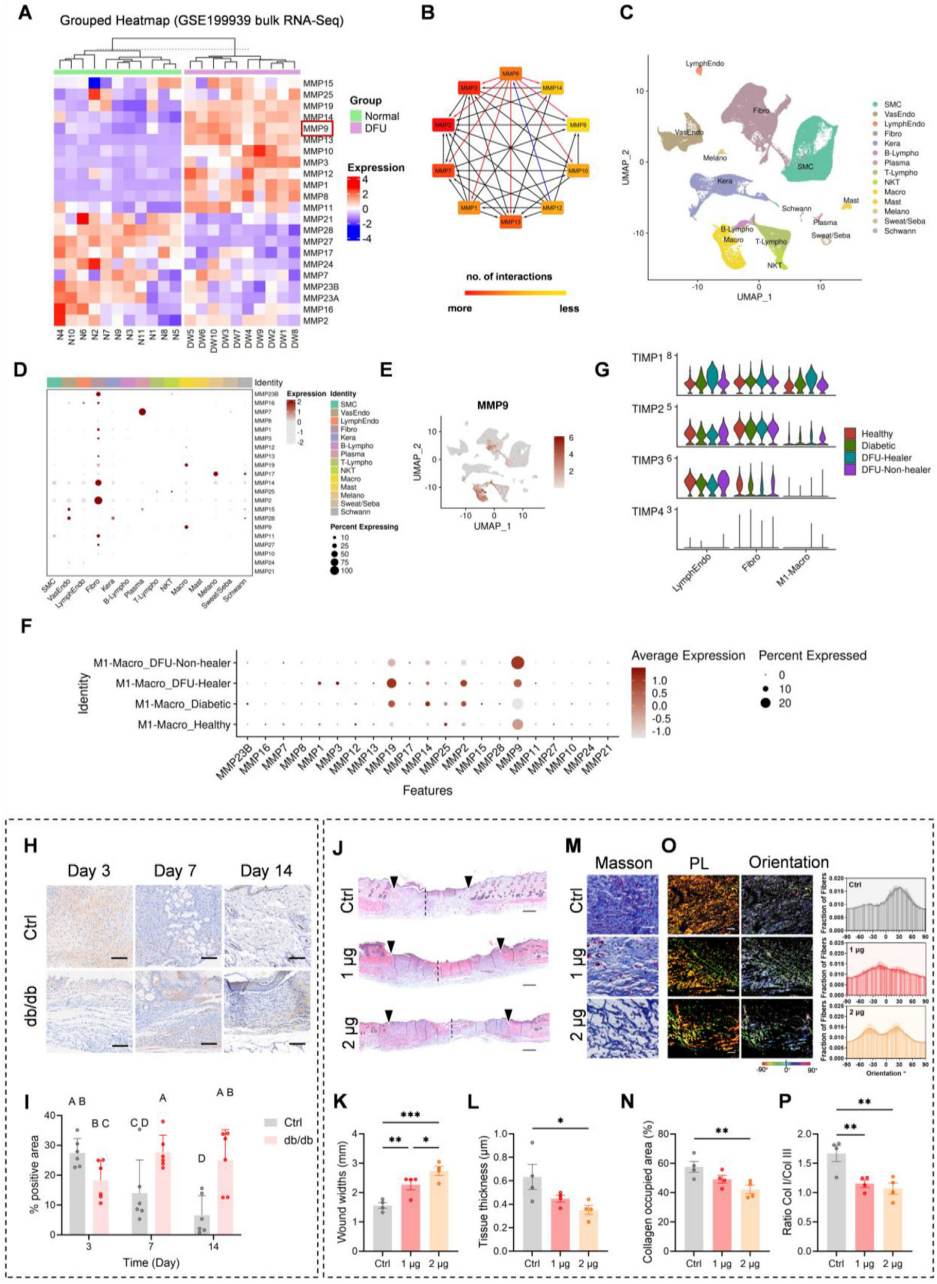
The role and expression characteristics of MMP9 in Wound Healing. (A) Heatmap showing the expression of MMP genes in healthy donors and DFU patients. Data were retrieved and analyzed from GSE199939. (B) PPI network of interacting MMP proteins. The network was retrieved from the STRING database. Interacting MMP proteins are connected. The color of the nodes indicates the number of interactions. (C) UMAP of the scRNA‐Seq data of foot skin samples from healthy donors and foot ulcer samples from DFU patients. Data were retrieved from GSE165816. (D) Dot plot showing the expression of MMP genes in different cell types, as shown in (C). (E) UMAP showing the expression of MMP9. (F) Dot plot showing expression of all detected MMP genes in M1‐macrophage split by 4 patient groups. The percentage of cells expressed and the expression level are normalized within the 4 patient groups. (G) Violin plots showing expression levels of TIMP genes across different cell types (LymphEndo, Fibro, and M1‐Mcro) and clinical groups. (H) Representative images showing MMP9 expression in the wound regions of control and db/db mice on days 3, 7, and 14. Scale bars: 100 µm. (I) Quantification of MMP9‐positive area in the wound regions of control and db/db mice on day 3, day 7, and day 14. n = 6. (J) Representative images of H&E‐stained wounded skin from mice on day 7, with or without MMP9 protein injection. Black arrows indicate wound edges. The dotted line shows where granulation tissue thickness was measured. Scale bars: 2 mm. (K‐L) Quantification of wound width (J) and thickness of granulation tissue (K) in the wound regions of different groups. n = 4. (M) Representative images of Masson's trichrome‐stained wounded skin from mice on day 7 with or without MMP9 protein injection. Scale bars: 50 µm. (N) Quantification of collagen‐positive area in the wound regions of different groups. n = 4. (O) Representative images of birefringent collagen in the wound region (left) and color‐coded map of collagen fiber orientation (middle) from different groups. 0° corresponds to the horizontal axis of the image. The histogram (right) showed the distribution of associated collagen fiber orientations. The orientation analysis was performed using OrientationJ software. Scale bars: 50 µm. (P) Quantification of the ratio of Col I/Col III as shown in Sirius Red‐stained wounded skin (left). n = 4. Data are shown as mean ± s.e.m.; Two‐way ANOVA with Fisher's Least Significant Difference (LSD) Test (H); One‐way ANOVA with Fishers’ LSD test (J, K, M, O); *p* < 0.05, ^**^ *p* < 0.01, and ^***^ *p* < 0.001.

To identify the key MMP enzymes underlying impaired healing in diabetic wounds, we first performed bioinformatic analyses of MMP gene expression profiles in DFUs. Differential expression analysis of the GSE199939 dataset revealed distinct expression patterns for multiple MMP family members in DFU wound tissues compared to healthy skin tissues (Figure 1A). Protein‐protein interaction (PPI) network analysis of MMP interactions revealed that MMP‐9 is located in a core node, interacting with 8 MMP family members (excluding MMP12), suggesting a pivotal role in the MMP family regulatory network (Figure 1B). Uniform Manifold Approximation and Projection (UMAP) clustering analysis of the single‐cell RNA sequencing dataset GSE165816 demonstrated that MMP‐9 is mainly localized in macrophages, and the expression level of MMP‐9 in M1 macrophages of the DFU‐Non‐healer group was significantly higher than that in other tissue groups (Healthy, Diabetic, and DFU‐healer) (Figure 1C–F; Figure S1A,B). In addition, as an endogenous inhibitor of the MMP family, the expression level of TIMP1 in M1 macrophages of DFU patients with successful healing was significantly higher than that of DFU patients with poor healing, implying that MMP9/TIMP balance dysregulation may be involved in the regulation of diabetic wound healing (Figure 1G).

The characteristics of MMP‐9 expression were further verified in a diabetic wound healing model. Immunohistochemical analysis indicated that MMP‐9 expression in wild‐type mouse wound tissues peaked on day 3 and declined on days 7 and 14 (Figure 1H,I). In contrast, the percentage of MMP‐9‐positive area in db/db mouse wound tissues remained consistently high (all ≥20%), reaching 26% and 25% on days 7 and 14, respectively (Figure 1H,I). To assess the functional impact of MMP‐9 on wound healing and simulate the pathological high‐MMP‐9 microenvironment observed in diabetic wounds, 1 µg and 2 µg of MMP‐9 were injected into the wound healing model of wild‐type mice. Histological analysis on day 7 demonstrated that MMP‐9 impaired wound healing in a concentration‐dependent manner. Compared with the control group, the MMP‐9‐treated groups exhibited significantly increased wound width and decreased granulation tissue thickness (Figure 1J–L). Collagen deposition was reduced, and collagen fibers were disorganized in the MMP‐9‐injected groups (Figure 1M–O). MMP‐9 injection also decreased the type I collagen (Col I)/type III collagen (Col III) ratio (Figure 1P). Collectively, these findings confirm that abnormally high MMP‐9 expression in the wound microenvironment is closely associated with delayed wound healing and extracellular matrix (ECM) disorganization, indicating that MMP‐9 is a critical therapeutic target for regulating diabetic wound healing.

### 2.2 The M9RR System Enables MMP‐9‐Dependent Release of Target Proteins

**FIGURE 2:**
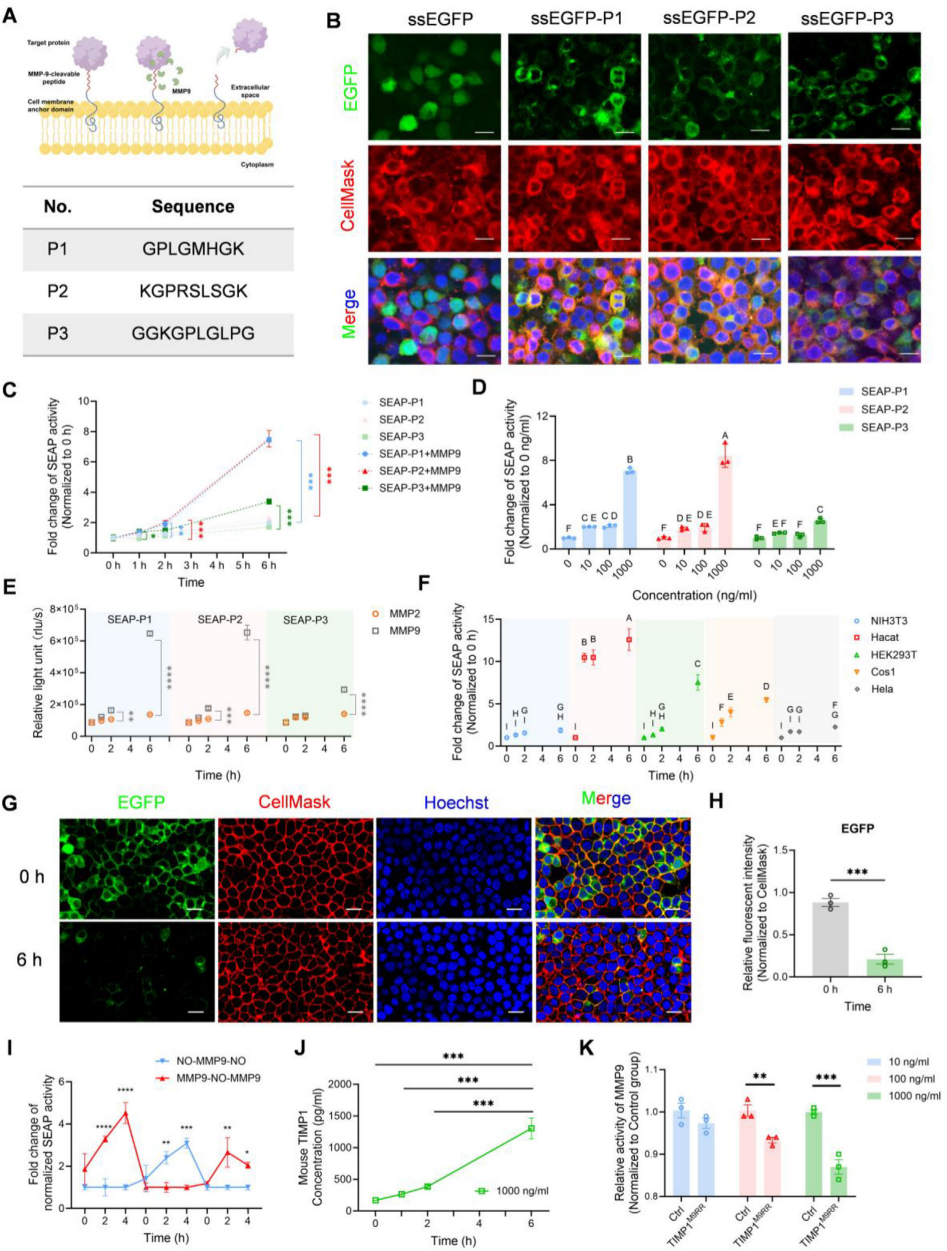
Characterization of the MMP9‐responsive protein release system. (A) Schematic illustration of TIMP1 membrane anchoring and three candidate cleavage peptide sequences (by FigDraw). (B) Representative confocal microscopy images showing the cellular localization of the GPI anchor and three candidate peptide sequences fused to ssEGFP (EGFP with secretion signals): ssEGFP (free ssEGFP), ssEGFP‐P1/P2/P3 (ssEGFP fused with three candidate peptide sequences). CellMask (red, cell membrane); Hoechst 33342 (blue, cell nucleus). Scale bars: 20 µm. (C) Detection of SEAP activity from three SEAP‐peptide fusion proteins with or without MMP9 (1000 ng/ml) treatment at the indicated time points. Statistical analyses were performed between the MMP9‐treated group and the untreated control for each fusion protein at each time point. n = 3. (D) Detection of SEAP activity from three SEAP‐peptide fusion proteins at 6 h post‐treatment with different concentrations of MMP9. Statistical analysis was performed among different concentrations of MMP9 for each fusion protein. n = 3. (E) Comparison of the responsiveness of the three SEAP‐peptide fusion proteins to MMP2 and MMP9 enzymes at the indicated time points. Statistical analysis was performed between the MMP2‐treated group and the MMP9‐treated group for each fusion protein at each time point. n = 3. (F) Detection of SEAP activity at different time points in various cell lines after transfection with SEAP‐P2 fusion protein. Statistical analyses were performed among different time points for each cell line. n = 3. (G) Representative confocal microscopy images of ssEGFP‐P2 fusion protein localization following 6 h of MMP9 treatment. CellMask (red, cell membrane), Hoechst 33342 (blue, cell nucleus). Scale bars: 20 µm. (H) Quantitative analysis of ssEGFP fluorescence intensity at the cell membrane. The fluorescence intensity was normalized to the signal intensity on the cell membrane. n = 3 independent experiments. (I) Dynamic changes in SEAP activity in the supernatant following periodic MMP9 treatment of cells transfected with SEAP‐P2 fusion protein. The SEAP activity of the MMP9 treatment group was normalized to that of the corresponding non‐MMP9 treatment group. n = 3. (J) Detection of TIMP1 concentration in the supernatant from cells transfected with TIMP1‐P2 fusion protein at the indicated time points after treatment with MMP9. n = 3. (K) Detection of MMP9 activity in the culture medium using a fluorescent substrate of MMP‐9 (MOCAc‐PLGL(Dpa)AR). Control and TIMP1‐P2 fusion protein (TIMP1^M9RR^)‐transfected cells were incubated in the medium containing the indicated concentrations of MMP9 for 12 h. n = 3. Data are presented as mean ± s.e.m.; Two‐way ANOVA with Fisher's LSD test (C, D, E, F, I, K); Unpaired T‐test (H); One‐way ANOVA with Fisher's LSD test (J); ns, not significant, ^*^ *p* < 0.05, ^**^ *p* < 0.01, and ^***^ *p* < 0.001; Values sharing the same letter are not significantly different (p > 0.05).

The M9RR system is designed as a membrane‐anchored fusion protein, where an MMP‐9‐sensitive peptide links the C‐terminus of target proteins [secretion signal‐fused EGFP (ssEGFP), secreted alkaline phosphatase (SEAP), TIMP1] to the N‐terminus of a cell membrane‐anchoring domain previously reported to anchor secretory proteins extracellularly (Figure 2A) [18]. Under conditions of elevated extracellular MMP‐9, its proteolytic activity specifically cleaves the linking peptide, releasing the target protein from the cell membrane into the extracellular matrix (ECM) (Figure 2A).

To identify the optimal MMP‐9‐sensitive peptide sequence, we performed screening experiments in HEK293T cells. Confocal laser scanning microscopy (CLSM) images showed that all three candidate peptides successfully anchored ssEGFP to the cell membrane (Figure 2B). In time‐dependent response experiments, cells transfected with plasmids expressing SEAP‐GPI fusions linked by three different peptides exhibited a gradual increase in SEAP release upon active MMP‐9 stimulation (Figure 2C; Figure S2A). At 6 h post‐MMP‐9 stimulation, SEAP activity in the supernatant of SEAP‐P1, P2, and P3 groups was significantly higher than that in their respective untreated controls (Figure 2C). Dose‐dependent response analysis revealed that stimulation with 1000 ng/ml MMP‐9 increased SEAP activity in the SEAP‐P2 group by approximately eightfold compared to the 0 ng/ml group, indicating that P2 has superior MMP‐9 responsiveness compared to P1 and P3 (Figure 2D).

MMP‐2 and MMP‐9 belong to the gelatinase subgroup of MMPs and share similar substrates [19]. To evaluate the substrate specificity of the M9RR system for MMP‐9, fusion proteins were treated with MMP‐2. All fusion protein groups exhibited significantly higher responsiveness to MMP‐9 than to MMP‐2, confirming the system's MMP‐9 substrate specificity (Figure 2E). Collectively, P2 was identified as the optimal sensitive peptide and was used to construct all subsequent M9RR systems.

Next, the efficacy and reliability of the M9RR system were validated in various mammalian cell lines, including mouse embryonic fibroblasts (NIH/3T3), human immortalized keratinocytes (HaCaT), human embryonic kidney cells expressing the SV40 large T antigen (HEK293T), African green monkey kidney fibroblasts (COS1), and human cervical cancer cells (HeLa). In all cell types, elevated MMP‐9‐responsive SEAP activity was detected in the supernatant of all cell lines at 6 h post‐treatment, demonstrating the broad applicability of the M9RR system in mammalian cells (Figure 2F). Immunofluorescence analysis was performed to visualize MMP‐9‐responsive protein release. CLSM images showed that, in cells transfected with the ssEGFP‐P2 construct, the EGFP fluorescence intensity predominantly localized at the cell membrane was significantly reduced after 6 h of MMP‐9 treatment, confirming efficient protein release by the system (Figure 2G,H).

To assess the reversibility of the M9RR system, cells transfected with the SEAP‐P2 plasmid were exposed to cyclic changes in MMP‐9 concentrations (0 and 1000 ng/ml) over three 4‐h intervals. As expected, SEAP activity in the supernatant exhibited periodic increases and decreases, confirming that the system responds dynamically to fluctuating MMP‐9 concentrations (Figure 2I).

Finally, we engineered the TIMP1^M9RR^ system by incorporating TIMP1 as the therapeutic target protein. MMP‐9 exposure experiments confirmed that TIMP1 was efficiently released within 6 h (Figure 2J). To verify the biological activity of the released TIMP1, TIMP1^M9RR^‐transfected cells were treated with different concentrations of MMP‐9, and the residual MMP‐9 activity in the supernatant was measured using an MMP‐9 fluorescent substrate. As anticipated, higher MMP‐9 concentrations stimulated increased TIMP1 release, which neutralized more MMP‐9 molecules, leading to a greater reduction in supernatant MMP‐9 activity (Figure 2K). In addition, Cell Counting Kit‐8 (CCK‐8) assays, 5‐ethynyl‐2'‐deoxyuridine (EdU) fluorescent imaging, and flow cytometry analysis showed that TIMP1^M9RR^ transfection did not significantly affect HEK293T cell proliferation (Figure S2B–E).

Collectively, these data establish that the M9RR system exhibits MMP‐9‐dependent responsiveness, MMP‐9 substrate specificity, broad applicability across various mammalian cell lines, and response reversibility. Importantly, TIMP1 released by the TIMP1^M9RR^ system effectively neutralized MMP‐9 activity in a concentration‐dependent manner.

### 2.3 The TIMP1^M9RR^ System Protects Skin Cells Against MMP‐9‐Induced Damage

As one of the best‐characterized secretory endopeptidases, MMP‐9 plays a critical role in extracellular matrix (ECM) remodeling, cell migration, angiogenesis, and immune responses [20]. To evaluate the protective effect of the TIMP1^M9RR^ system against MMP‐9‐induced cell damage, HaCaT keratinocytes and BJ fibroblasts were used as in vitro models. TIMP1^M9RR^ was delivered via SM102 lipid nanoparticles (LNPs), and multiple functional assays were performed to assess the regulatory effects of the TIMP1^M9RR^ system on cell function following MMP‐9‐induced injury.

**FIGURE 3:**
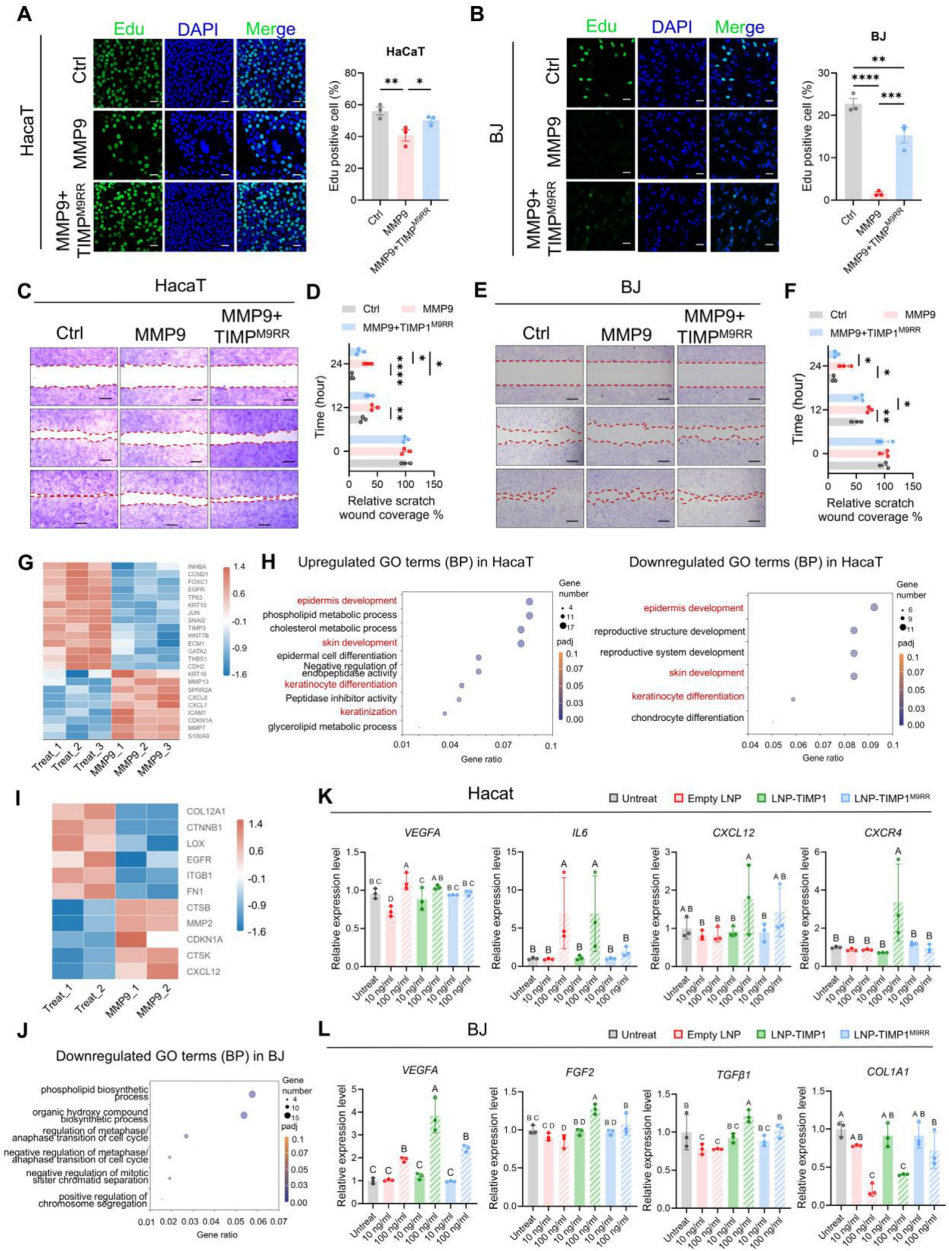
On‐demand TIMP1 delivery rescues MMP9‐induced impairment in vitro. (A) Representative images (left) and quantification analysis (right) of the EdU assay in control, MMP9, and MMP9+ TIMP1^M9RR^ HaCaT cell groups. Scale bars: 40 µm; n = 3. (B) Representative images (left) and quantification analysis (right) of the EdU assay in control, MMP9, and MMP9+ TIMP1^M9RR^ BJ cell groups. Scale bars: 40 µm; n = 3. (C) Representative images of the scratch‐wound assay in control, MMP9, and MMP9+ TIMP1^M9RR^ HaCaT cell groups. Scale bars: 200 µm. (D) Quantitative analysis of the scratch wound coverage in different HaCaT cell groups. Statistical analysis was performed in different groups at each time point. n = 3. (E) Representative images of the scratch‐wound assay in control, MMP9, and MMP9+ TIMP1^M9RR^ BJ cell groups. Scale bars: 200 µm. (F) Quantitative analysis of the scratch wound coverage in different BJ cell groups. Statistical analysis was performed in different groups at each time point. n = 3. (G) Heatmap of representative differentially expressed genes in different HaCaT samples. Colors represent relative expression levels (hotter colors indicate higher expression). (H) GO analyses of the upregulated (left) and downregulated (right) biological processes (BP) in MMP9+ TIMP1^M9RR^‐treated HaCaT groups compared to MMP9‐treated HaCaT samples. (I) Heatmap of the representative differentially expressed genes in different BJ samples. Colors represent relative expression levels (hotter colors indicate higher expression). (J) GO analyses of the downregulated biological processes (BP) in MMP9+ TIMP1^M9RR^‐treated BJ groups compared to MMP9‐treated BJ samples. (K‐L) qPCR analysis of target gene expression in HaCaT (K) or BJ (L) cells under different treatments. Cells were either transfected (control) or transfected with empty lipid nanoparticles (Empty LNP), LNP‐TIMP1, or LNP‐TIMP1^M9RR^, followed by a 24‐h treatment with MMP9 at the indicated concentrations n = 3. Data are shown as mean ± s.e.m.; One‐way ANOVA with Fisher's LSD test (A, B, K, L); Two‐way ANOVA with Fisher's LSD test (D, F). *P* < 0.05, ^**^ *p* < 0.01, and ^***^ *p* < 0.001; values sharing the same letter are not significantly different (p > 0.05).

First, we confirmed the efficiency and persistence duration of mRNA delivery. Immunofluorescence imaging showed that eGFP mRNA encapsulated in SM102 LNPs sustained expression for at least 5 days post‐transfection (Figure S3A,B). EdU proliferation assays revealed that MMP‐9 treatment significantly reduced the EdU‐positive rate in HaCaT and BJ cells compared with the untreated control group. In contrast, the TIMP1^M9RR^‐treated group exhibited a significant increase in EdU‐positive cells (Figure 3A,B). Furthermore, cell scratch wound assays demonstrated that MMP‐9 exposure significantly impaired the migratory capacity of both HaCaT and BJ cells, whereas TIMP1^M9RR^ treatment partially reversed this inhibitory effect (Figure 3C–F).

To further elucidate the molecular mechanism underlying the protective effect of TIMP1^M9RR^ against MMP‐9‐induced damage, transcriptome analysis was performed (Figure S3C,D). In HaCaT cells, key differentially expressed gene (DEG) analysis showed that the TIMP1^M9RR^ system targeted the expression of genes involved in matrix remodeling (e.g., *MMP13* and *MMP7*), cell fate determination (e.g., *TP63* and *KRT10*), inflammatory responses (e.g., *CXCL1* and *CXCL8*), and cell adhesion (e.g., *SNAI2*), thereby alleviating MMP‐9‐induced perturbations in HaCaT cell behavior (Figure 3G). Gene Ontology (GO) functional enrichment analysis (P<0.01) indicated that genes upregulated by TIMP1^M9RR^ were significantly associated with keratinocyte differentiation and epidermal development (Figure 3H). In contrast, downregulated genes were also enriched in overlapping pathways, such as epidermal development and skin development, suggesting that TIMP1^M9RR^ is involved in precisely regulating HaCaT cell differentiation by balancing gene expression in these pathways (Figure 3H). In BJ fibroblasts, key DEG analysis revealed that MMP‐9 regulated the expression of genes associated with cell adhesion (e.g., *FN1* and *ITGB1*), matrix remodeling (e.g., *MMP2* and *COL12A1*), proliferation (e.g., *ITGB1* and *CTNNB1*), and inflammatory responses (e.g., *CXCL12*) (Figure 3I). However, GO functional enrichment analysis for biological processes (BP) in BJ cells identified downregulated pathways, including phospholipid biosynthetic process, organic hydroxy compound biosynthetic process, and regulation of metaphase/anaphase transition of the cell cycle (Figure 3J). Collectively, these results demonstrate that the TIMP1^M9RR^ system mitigates MMP‐9‐induced damage by regulating the expression of DEGs core to cell function.

Finally, to further evaluate the protective effects of TIMP1^M9RR^ against damage induced by different concentrations of MMP‐9, HaCaT and BJ cells transfected with TIMP1^M9RR^ were treated with two concentrations of MMP‐9 (10 and 100 ng/ml). After 24 h, the expression levels of several inflammatory cytokines and growth factors known to be secreted by these cells were measured [21, 22, 23]. Results showed that transfection with TIMP1 (either LNP‐TIMP1 or LNP‐ TIMP1^M9RR^) partially alleviated the MMP‐9‐induced alterations in the expression of vascular endothelial growth factor‐A (*VEGFA*) and interleukin‐6 (*IL6*) in HaCaT cells, as well as transforming growth factor‐beta 1 (*TGFB1*) and fibroblast growth factor 2 (*FGF2*) in BJ cells (Figure 3K,L). Notably, LNP‐TIMP1^M9RR^ exhibited expression profiles most similar to the untreated group across different MMP‐9 concentrations, whereas LNP‐TIMP1 exhibited pronounced deviations with increasing or decreasing MMP‐9 concentrations (Figure 3K,L). These findings confirm that the on‐demand release of TIMP1 by the TIMP1^M9RR^ system protects HaCaT and BJ from MMP‐9‐induced dysfunction under variable pathological conditions.

### 2.4 The TIMP1^M9RR^ System Significantly Improved Wound Healing in db/db Diabetic Mice

**FIGURE 4:**
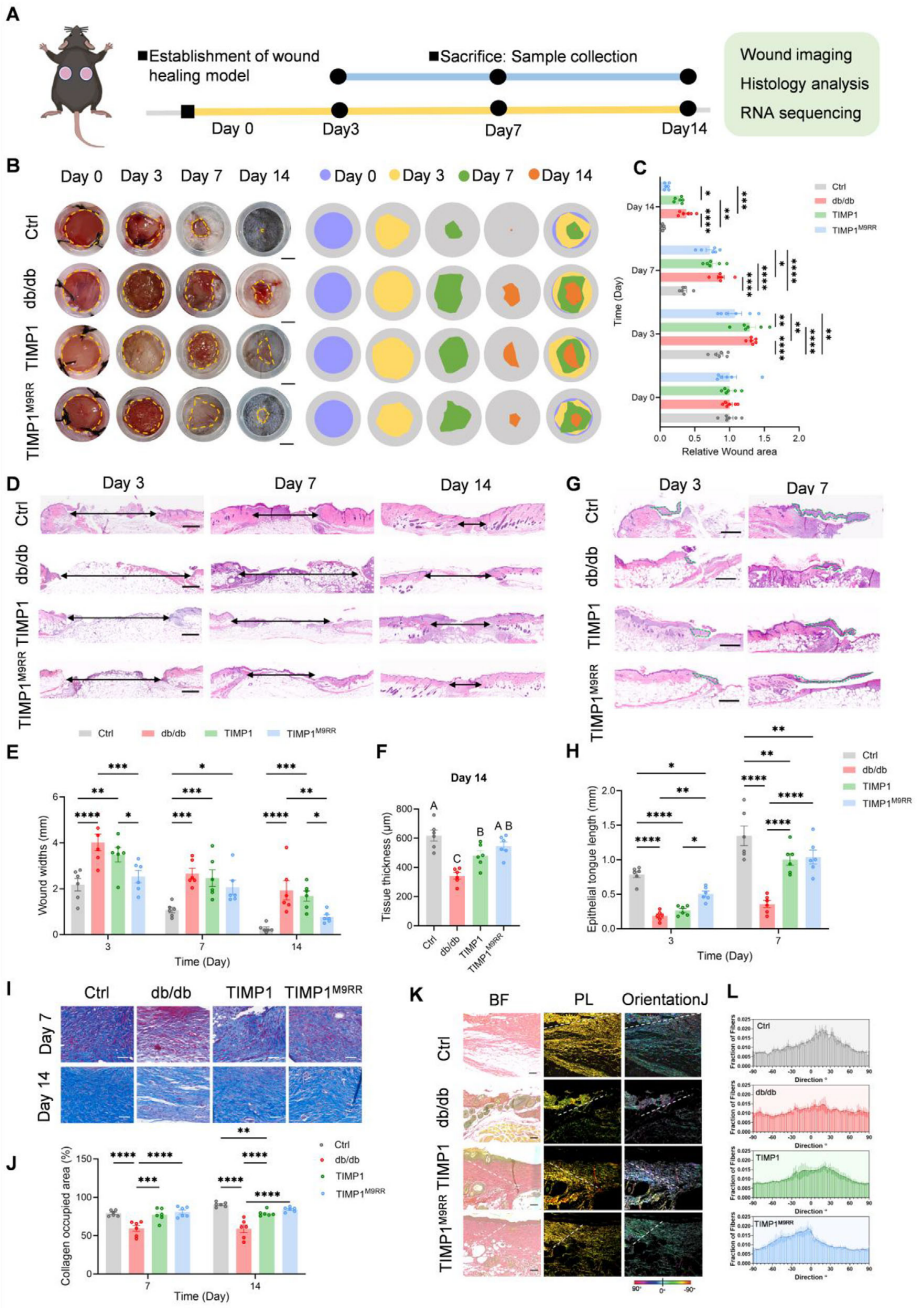
TIMP1^M9RR^ promotes wound healing in db/db mice. (A) Timeline for in vivo experiments in db/db mice. (B) Gross view (left) and simulation plots of the wound closure areas (right) of wounds from mice in different groups as indicated on days 0, 3, 7, and 14 post‐surgery. Scale bars: 2 mm. (C) Quantitative analysis of wound closure rates over time for each group. n = 6. (D) Representative images of H&E staining of wound sections in different groups at the indicated time points. Scale bar: 500 µm. (E‐F) Quantitative measurements of wound width (D) and granulation tissue thickness (E). n = 6. (G) High‐magnification images showed epithelial tongues in different groups on day 3 and day 7. Scale bars: 250 µm. (H) Quantitative analysis of epithelial tongue length. n = 6. (I) Representative images of Masson's trichrome staining of wound sections in different groups at the indicated time points. Scale bar: 50 µm. (J) Quantification analysis of the collagen‐occupied area percentage. n = 6. (K) Representative images of Sirius Red‐stained wounded skin (left), birefringent collagen under polarized light (middle), and color‐coded map of collagen fiber orientation (right) from different groups. BF, bright field; PL, polarized light; 0° corresponds to the horizontal axis of the image. Scale bars: 100 µm. (L) Representative histogram showing the distribution of collagen fiber orientations. The orientation analysis was performed using OrientationJ software. Data are shown as mean ± s.e.m.; Two‐way ANOVA with Fisher's LSD test (C, E, H, J); One‐way ANOVA with Fisher's LSD test (F); *p* < 0.05, ^**^ *p* < 0.01, and ^***^ *p* < 0.001.

To evaluate the in vivo therapeutic efficacy of the TIMP1^M9RR^ system, a wound healing model was established in db/db diabetic mice (Figure 4A). In vivo imaging confirmed that local and efficient gene expression was achieved as early as 1 day after subcutaneous injection of SM102 LNP‐mediated mRNA delivery, and persisted for up to 7 days postoperatively (Figure S4A,B). Immunohistochemical (IHC) analysis of TIMP1 revealed that the percentage of TIMP1‐positive area in wound tissues of db/db mice was significantly lower than that in control mice on days 3 and 7 (Figure S4C,D). In contrast, TIMP1 delivery (either TIMP1 or TIMP1^M9RR^) significantly increased the proportion of TIMP1‐positive area compared to the db/db group (Figure S4C,D). Notably, the TIMP1‐positive area in the TIMP1^M9RR^ group showed a time‐dependent increase on day 3, whereas no statistical difference was observed between days 3 and 7 in the TIMP1 group. This phenomenon may be attributed to the time‐dependent elevation of MMP‐9 expression in db/db mouse wounds, which induces the on‐demand release of more TIMP1 from the TIMP1^M9RR^ system (Figure 1G,H, Figure S4C,D). Consistently, ELISA quantification of wound tissue homogenate supernatant further validated this regulatory pattern (Figure S4E,F). The db/db diabetic wounds maintained persistently elevated MMP‐9 protein concentrations on day 7 and day 14, which were statistically higher than those in the normal control group. For the TIMP1 protein, the TIMP1 treatment group exhibited constitutive TIMP1 secretion, with comparable tissue TIMP1 levels detected between day 3 and day 7. In contrast, the TIMP1^M9RR^ group showed higher tissue TIMP1 abundance than the control group on day 7 but not on day 3, a trend that closely paralleled the progressive accumulation of MMP‐9 within diabetic lesions.

Macroscopic assessment and wound area quantification revealed progressive healing with continuous contraction in controls on days 3, 7, and 14. In contrast, db/db mice wounds slightly expanded early (day 3), then contracted but remained significantly larger than controls at all time points (Figure 4B,C). Both TIMP1‐delivery groups improved healing, with the TIMP1^M9RR^ group showing more pronounced wound contraction on days 3 and 14, demonstrating a distinct healing advantage (Figure 4B,C). Hematoxylin and eosin (H&E) staining revealed significantly wider wounds in db/db mice compared to controls at all healing stages (Figure 4D,E). TIMP1 delivery reduced wound width, with TIMP1^M9RR^ exerting a more potent effect than TIMP1 on days 3 and 14 (Figure 4D,E). Given that MMP‐9 degrades the dermal extracellular matrix (ECM) [10, 19], we hypothesized that reducing MMP‐9 activity in the wound would facilitate granulation tissue accumulation. Quantitative analysis of central granulation thickness revealed increased thickness in both TIMP1 groups on day 14 compared to db/db mice (Figure 4F). Furthermore, H&E‐based evaluation of epithelial tongue length (days 3, 7) showed superior regeneration in both TIMP1 treatment groups compared to the db/db group, with TIMP1^M9RR^ exerting a more pronounced effect on epithelial regeneration than TIMP1 on day 3 (Figure 4G,H).

Collagen is a major substrate of MMP‐9 [24]; therefore, reducing active MMP‐9 is expected to increase collagen content. Masson's trichrome staining showed greater wound collagen deposition in both TIMP1 treatment groups than in db/db mice on days 7 and 14 (Figure 4I,J). To further evaluate collagen accumulation, Sirius Red staining was performed on day 14. Under polarized light microscopy, early‐stage Col III displayed blue‐green birefringence while mature Col I showed orange‐yellow birefringence, enabling differentiation and quantification of the two subtypes [25]. In the control group, thick orange‐yellow collagen bundles were prominent in the newly formed tissue, while db/db wounds contained only sparse green‐yellow fibers. Notably, mice receiving the two TIMP1‐based therapeutic regimens exhibited a collagen profile closely resembling that of the control group (Figure 4K). Furthermore, analysis of collagen fiber orientation at the wound edges showed tightly arranged, regular collagen fibers extending from the uninjured region into the wound bed in the control group (Figure 4L). In the db/db group, however, few and disorganized collagen fibers extended from the uninjured region into the granulation tissue (Figure 4L). Both TIMP1 treatment groups improved fiber alignment, with patterns closely resembling those of the control group (Figure 4L). Collectively, these findings demonstrate that TIMP1, especially TIMP1^M9RR^, ameliorates impaired collagen remodeling in diabetic wounds and facilitates physiological tissue repair.

**FIGURE 5:**
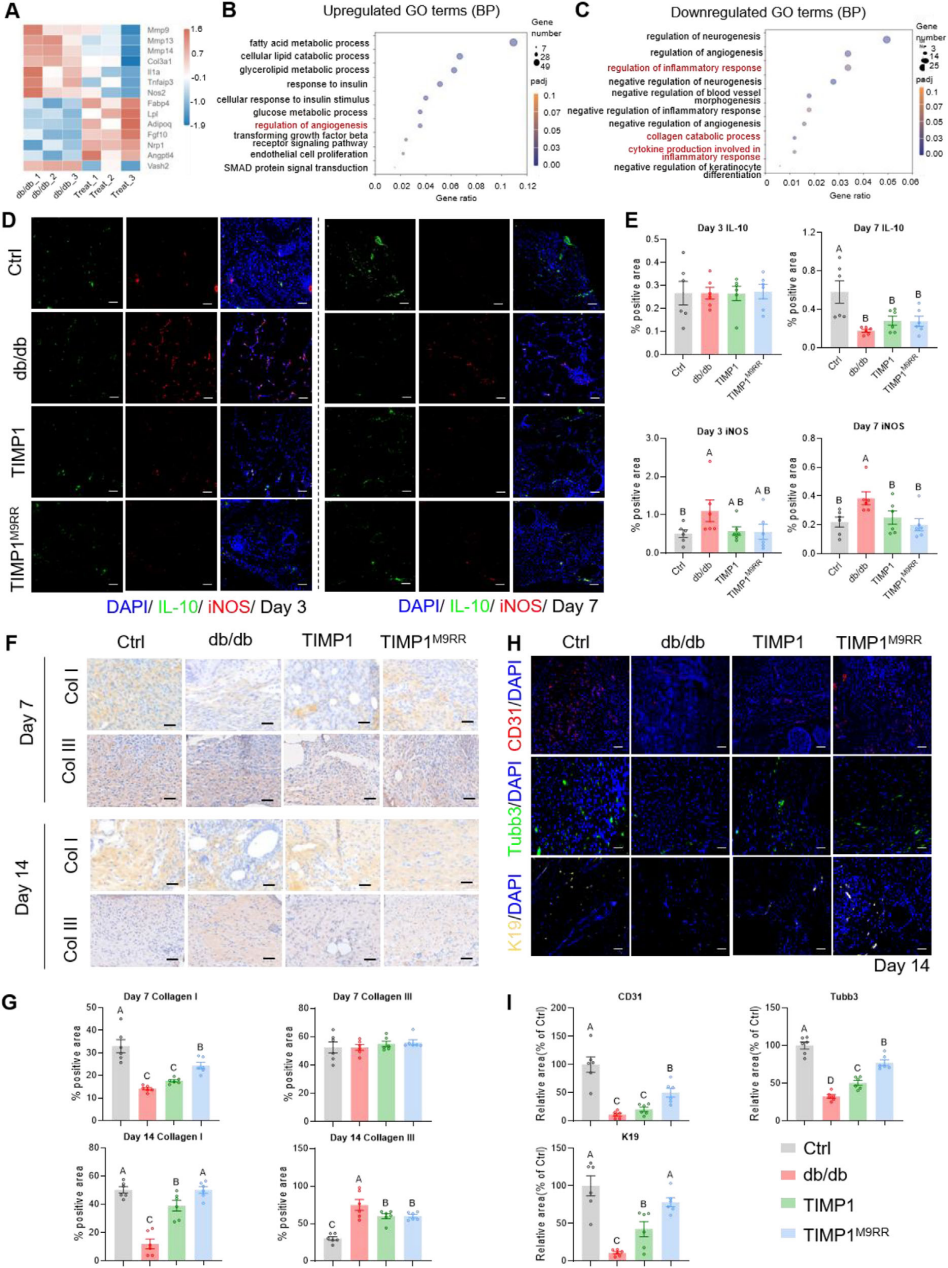
TIMP1^M9RR^ modulates immune response, collagen remodeling, and tissue regeneration in diabetic wounds. (A) Heatmap of the representative DEGs in different BJ samples. Colors represent relative expression levels (hotter colors indicate higher expression). (B‐C) GO analyses of the upregulated (B) and downregulated (C) biological processes (BP) in TIMP1^M9RR^ groups compared to untreated db/db samples. (D) Representative images showing the expression of IL‐10 (green) and iNOS (red) in wound regions from mice in different groups on day 3 and day 7. Nuclei are stained with Hoechst 33342 (blue). Scale bars: 50 µm. (E) Quantitative analysis of IL‐10 and iNOS positive area percentage on day 3 and day 7. n = 6. (F) Representative immunohistochemical images showing the expression of collagen I and collagen III in the wound region from mice in different groups on day 7 and day 14. Scale bars: 100 µm. (G) Quantitative analysis of collagen I and collagen III positive area percentage on day 7 and day 14. n = 6. (H) Representative immunofluorescent staining of CD31 (red), β3‐Tubulin (TUBB3, green), and Cytokeratin 18 (K19, yellow) in the wound regions of mice in different groups on day 14. Nuclei are visualized with Hoechst 33342 (blue). Scale bars: 50 µm. (I) Quantitative analysis of CD31, TUBB3, and K19 positive area percentage on day 14. n = 6. Data are shown as mean ± s.e.m. One‐way ANOVA with Fisher's LSD test (E, J, I). Values sharing the same letter are not significantly different (p > 0.05).

To further explore the potential mechanism by which TIMP1^M9RR^ promotes wound healing, transcriptome sequencing was performed on newly formed wound tissues on day 3 postoperatively (Figure S5A). Compared to the db/db group, the TIMP1^M9RR^‐treated wound showed significantly downregulated expression of pro‐inflammatory and ECM degradation‐related genes (e.g., *Il1a*, *MMP13*) and upregulation of genes involved in lipid metabolism and tissue repair (e.g., *Fabp4*, *Fgf10*) (Figure 5A). GO enrichment analysis further indicated that upregulated genes in the TIMP1^M9RR^ group were mainly enriched in pathways related to the regulation of glycolipid metabolism, insulin signaling, and angiogenesis, whereas downregulated genes were significantly enriched in pathways involved in the regulation of the inflammatory response and collagen degradation (Figure 5B). Integrated analysis across cell models (HaCaT and BJ) and the animal model identified 49 overlapping DEGs (Figure S5B,C). GO enrichment analysis showed that these overlapping DEGs were significantly enriched in skin‐related biological processes, particularly key pathways including epidermal differentiation, keratinocyte differentiation, skin barrier establishment, and regulation of water homeostasis (Figure S5D). Collectively, these transcriptomic findings suggest that TIMP1^M9RR^ promotes diabetic wound healing by coordinately regulating pathways associated with inflammation resolution, ECM remodeling, metabolic homeostasis, and skin barrier repair.

Given the prominent regulation of inflammation‐related pathways in the transcriptome analysis, we next investigated the expression of the anti‐inflammatory (IL‐10) and pro‐inflammatory (iNOS) markers at early‐ and mid‐stage wound‐healing time points. On day 3, the IL‐10 expression showed no significant differences among all groups (Figure 5D,E). On day 7, IL‐10 expression was significantly downregulated in the db/db group, while the TIMP1 and TIMP1^M9RR^ groups showed an upward trend (no statistical significance vs. db/db group). The inflammatory marker iNOS was significantly more highly expressed in the db/db group compared to the control group on day 3, and this high expression persisted until day 7 (Figure 5D,E). Notably, both TIMP1 treatment groups showed a downward trend in iNOS expression on day 3, which became more pronounced by day 7, with levels comparable to those in the control group. For the general pro‐inflammatory cytokine IL‐6, the db/db diabetic group exhibited persistently elevated IL‐6 average optical density at day 3 and day 14, significantly higher than the Ctrl group (Figure S5E,F). While two TIMP1 treatments moderately suppressed IL‐6 accumulation, the TIMP1^M9RR^ responsive group achieved a more robust inhibition of sustained IL‐6 overexpression during the late healing phase.

We further evaluated macrophage polarization by immunofluorescent staining of the M1 marker CD86 and the M2 marker CD206 (Figure S5G,H). On day 7, db/db wounds displayed markedly enhanced CD86 fluorescence intensity and reduced CD206 signals, indicating a dominant pro‐inflammatory M1 macrophage phenotype. Both TIMP1 intervention groups alleviated this imbalance.

Beyond the inflammatory modulation revealed by transcriptomic and immunofluorescence analyses, we further assessed the impact of TIMP1^M9RR^ on extracellular matrix (ECM) remodeling by quantifying Col I and Col III expression via immunohistochemistry (IHC). On days 7 and 14, the proportion of Col I‐expressing area in the db/db group was significantly lower than that in the control group (Figure 5F,G). TIMP1 delivery increased the proportion of Col I‐positive area, with a more pronounced upregulation observed in the TIMP1^M9RR^ group. In contrast, the percentage of Col III‐positive area was significantly higher in the db/db group than in the control group. Both TIMP1 treatment groups, however, showed a reduction in Col III‐positive area compared to the db/db model (Figure 5F,G).

We next evaluated myofibroblast activation by detecting α‐SMA expression on day 14 via IHC (Figure S5I,J). As shown in the quantitative AOD statistics (Figure S5J), α‐SMA relative optical density was significantly attenuated in db/db wounds relative to the control skin wound. After treatment with either TIMP1 or TIMP1^M9RR^, α‐SMA staining intensity was rescued, and no statistically significant differences were detected among the treatment groups and the control group.

Finally, markers of vascularization (CD31), neural regeneration (TUBB3), and hair follicle development (K19) were assessed on day 14. The positive areas for CD31, K19, and TUBB3 were all significantly diminished in the db/db group than in the control group. In contrast, delivery of TIMP1^M9RR^ partially restored these markers to levels close to those in the control group (Figure 5H,I).

Thus, these results confirmed that the TIMP1^M9RR^ system promotes comprehensive wound repair and regeneration in vivo.

### 2.5 CryoMNs Enable Efficient Cutaneous Gene Delivery and Enhance Target Protein Expression

**FIGURE 6:**
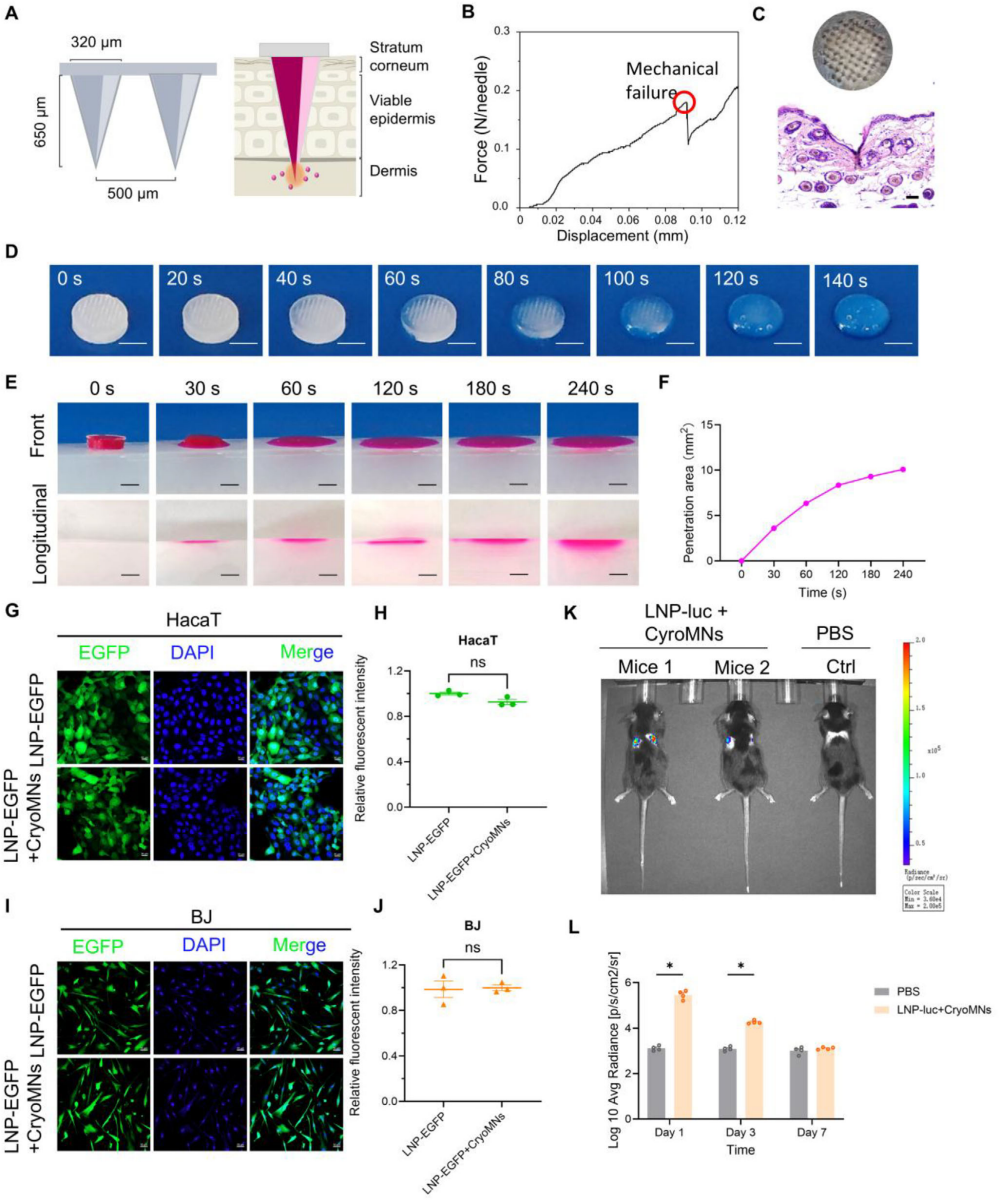
Fabrication and characterization of Cryo‐Microneedles (CryoMNs). (A) Schematic illustration of CryoMNs and their application mode to skin wounds. (B) The force‐displacement curve of CryoMNs loaded with bFGF and lipid nanoparticles‐TIMP1^M9RR^ (CryoMNs@bFGF/TIMP1^M9RR^). The red circle indicates the mechanical failure of the CryoMNs@bFGF/ TIMP1^M9RR^. (C) Gross view (up) and H&E staining image (down) of mouse skin with micropores formed after applying CryoMNs. Scale bars: 50 µm. (D) Melting behavior of CryoMNs at room temperature (25°C) over time. (E) Cargo penetration simulated by cryo‐microneedles loaded with Rhodamine B. Scale bars: 3 mm. (F) Quantitative analysis of the penetration area over time as shown in (E, down‐Longitudinal view). (G) Representative fluorescence images of EGFP in HaCaT cells transfected by lipid nanoparticles‐EGFP mRNA (LNP‐EGFP) and CryoMNs loaded with LNP‐EGFP (LNP‐EGFP+CryoMNs). Scale bars: 50 µm. (H) Quantitative analysis of transfection efficiency of LNP‐EGFP and LNP‐EGFP+CryoMNs in HaCaT cells. n = 3. (I) Representative fluorescence images of EGFP in BJ cells transfected with lipid nanoparticles‐EGFP mRNA (LNP‐EGFP) and CryoMNs loaded with LNP‐EGFP (LNP‐EGFP+CryoMNs). Scale bars: 50 µm. (H) Quantitative analysis of transfection efficiency of LNP‐EGFP and LNP‐EGFP+CryoMNs in BJ cells. n = 3. (K) In vivo imaging of C57 mice 24 h without and with application of CryoMNs loaded with lipid nanoparticles‐luciferase mRNA. (I) Comparison of average radiance [p/s/cm2/sr] between different groups. n = 4. Data are shown as mean ± s.e.m.; unpaired T‐test (H, J); multiple unpaired T‐test (L); ns, not significant, ^*^ *p* < 0.05, ^**^ *p* < 0.01, and ^***^ *p* < 0.001.

Microneedles (MNs) offer a promising strategy for enhancing diabetic wound therapy by combining minimally invasive delivery, enhanced transdermal penetration, and prolonged local drug retention [26]. To accommodate the thermolabile nature of mRNA [27], this study utilized cryoMNs (with a single‐needle height of 650 µm and a base diameter of 320 µm) as the delivery vector (Figure 6A). Mechanical testing revealed a breaking strength of 0.17 N per needle, well above the minimum force required for skin penetration (0.058 N per needle) [28], confirming that the cryoMNs possess sufficient mechanical strength to pierce human skin (Figure 6B). Penetration assays using wild‐type mouse skin further confirmed epidermal penetration, as evidenced by distinct needle tracks in H&E‐stained sections, with no significant damage to deeper dermal structures (Figure 6C). At room temperature, the cryoMNs dissolved completely within 140 s (Figure 6D). In addition, transdermal simulation in agarose gel using rhodamine B (RhB)‐loaded cryoMNs to mimic LNP diffusion revealed substantial RhB accumulation followed by lateral diffusion within the gel matrix (Figure 6E,F). To assess whether the microneedle matrix affected LNP transfection efficiency, we compared cryoMN‐encapsulated LNP‐EGFP mRNA with direct LNP‐mRNA delivery. Results indicated that encapsulating LNP‐EGFP mRNA in cryoMNs did not significantly compromise transfection efficiency compared with direct LNP‐mRNA delivery (Figure 6G–J). In vivo delivery experiments using LNP‐luciferase (LUC) mRNA showed that cryoMN‐mediated administration significantly increased LUC fluorescence intensity in the skin, which gradually returned to baseline by day 7 (Figure 6K,L). Similarly, cryoMNs delivery of TIMP1^M9RR^ mRNA LNPs enhanced TIMP1 expression in wounds by day 3 (Figure S6A,B). Collectively, these findings demonstrate that cryoMNs enable efficient, sustained, and localized cutaneous gene delivery.

### 2.6 Co‐Delivery of bFGF and TIMP1^M9RR^ via CryoMNs Enhances Diabetic Wound Healing

bFGF, a clinically used agent for wound healing, promotes healing by stimulating fibroblast and endothelial cell proliferation/migration, accelerating granulation tissue formation and angiogenesis, thereby shortening healing time [29, 30]. To investigate the potential impact of TIMP1^M9RR^ on the pro‐healing capacity of bFGF, we constructed a cryoMNs‐based co‐delivery system for wound repair in db/db mice.

**FIGURE 7:**
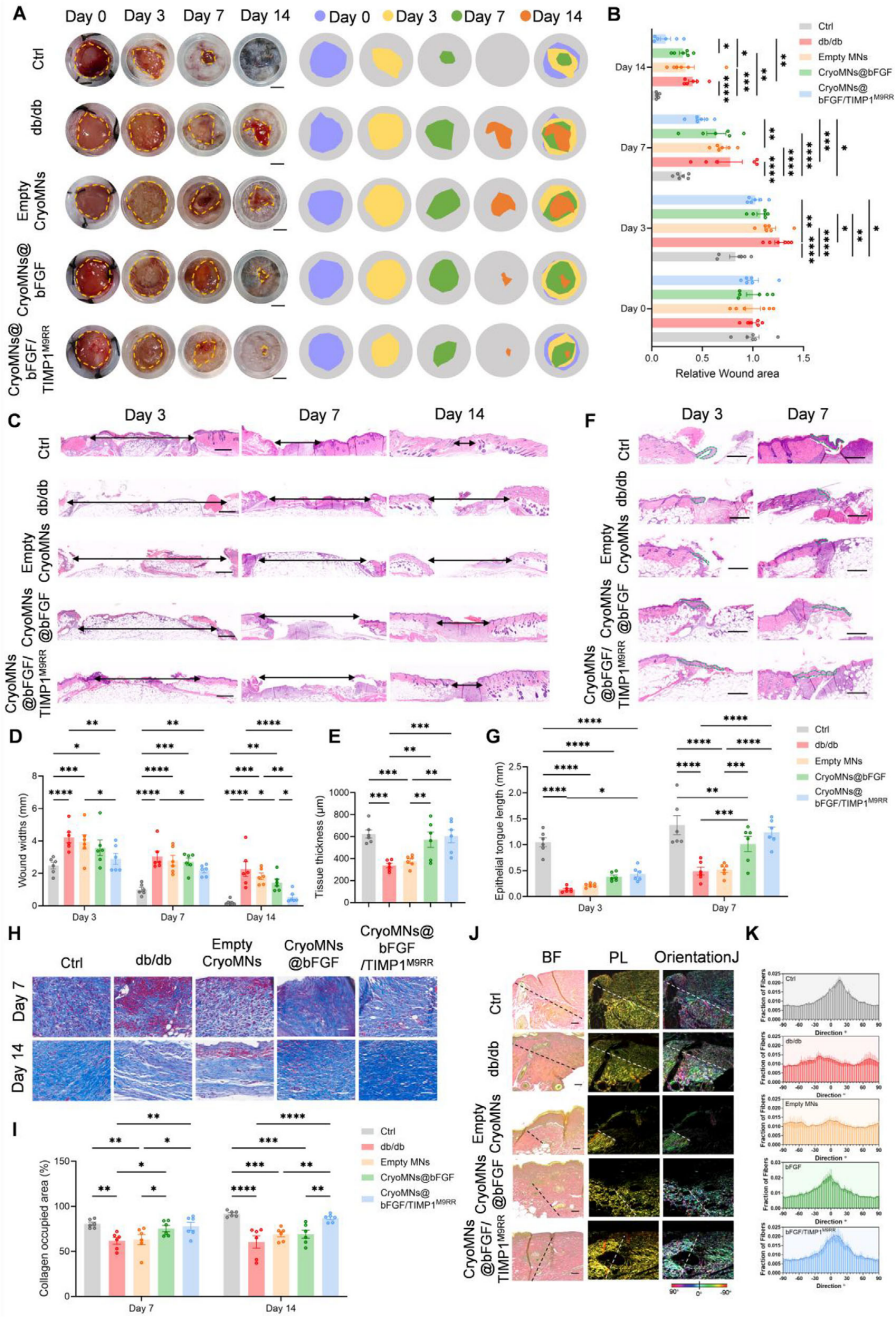
Both TIMP1^M9RR^ and bFGF enhance the therapeutic effect of bFGF on diabetic wound healing. (A) Gross view (left) and simulation plots of the wound closure areas (right) of wounds from mice in different groups on days 0, 3, 7, and 14 post‐surgery. Scale bars: 2 mm. (B) Quantitative analysis of wound closure rates over time for each group. n = 6. (C) Representative images of H&E staining of wound sections in different groups at the indicated time points. Scale bar: 500 µm. (D‐E) Quantitative measurements of wound width (D) and granulation tissue thickness (E). n = 6. (F) High‐magnification images showed epithelial tongues in different groups on day 3 and day 7. Scale bars: 250 µm. (G) Quantitative analysis of epithelial tongue length. n = 6. (H) Representative images of Masson's trichrome staining of wound sections in different groups at the indicated time points. Scale bar: 50 µm. (I) Quantification analysis of the collagen‐occupied area percentage. n = 6. (J) Representative images of Sirius Red‐stained wounded skin (left), birefringent collagen under polarized light (middle), and color‐coded map of collagen fiber orientation (right) from different groups. BF, bright field; PL, polarized light; 0° corresponds to the horizontal axis of the image. Scale bars: 100 µm. (K) Representative histogram showing the distribution of collagen fiber orientations. Orientation analysis was performed using OrientationJ software. Data are shown as mean ± s.e.m.; Two‐way ANOVA with Fisher's LSD test (B, D, G, I); One‐way ANOVA with Fisher's LSD test (E); *p* < 0.05, ^**^ *p* < 0.01, and ^***^ *p* < 0.001.

Macroscopic observation and wound area quantification showed that the therapeutic groups (CryoMNs@bFGF and CryoMNs@bFGF/TIMP1^M9RR^ groups) exhibited significantly improved wound healing compared to the empty cryoMNs group and the db/db group (Figure 7A,B). On day 14, the wound area in the CryoMNs@bFGF/ TIMP1^M9RR^ group was markedly smaller than that in the CryoMNs@bFGF group, with a healing status closer to the control group. H&E staining further confirmed the therapeutic effect of both bFGF and TIMP1 ^M9RR^. On day 14, the wound width in the CryoMNs@bFGF/ TIMP1^M9RR^ group was significantly smaller than that in the db/db, empty cryoMNs, and CryoMNs@bFGF groups (Figure 7C,D). Furthermore, both therapeutic groups had significantly greater granulation tissue thickness and longer epithelial tongue lengths compared to the db/db and empty CryoMNs groups (Figure 7E–G).

Masson's trichrome staining indicated enhanced collagen accumulation in both therapeutic groups, and by day 14, co‐delivery of bFGF and TIMP1^M9RR^ restored collagen deposition to a level comparable to the control group (Figure 7H,I). Sirius Red staining showed that, compared to the db/db and empty cryoMNs groups, the integration of collagen fibers extending from the unwounded area into the granulation tissue was significantly improved in both microneedle treatment groups (Figure 7J). Collagen fiber orientation at the wound site showed disorganized collagen fibers in the db/db and empty cryoMNs groups, whereas a relatively regular arrangement was observed in the control group and both cryoMNs therapeutic groups (Figure 7K).

**FIGURE 8:**
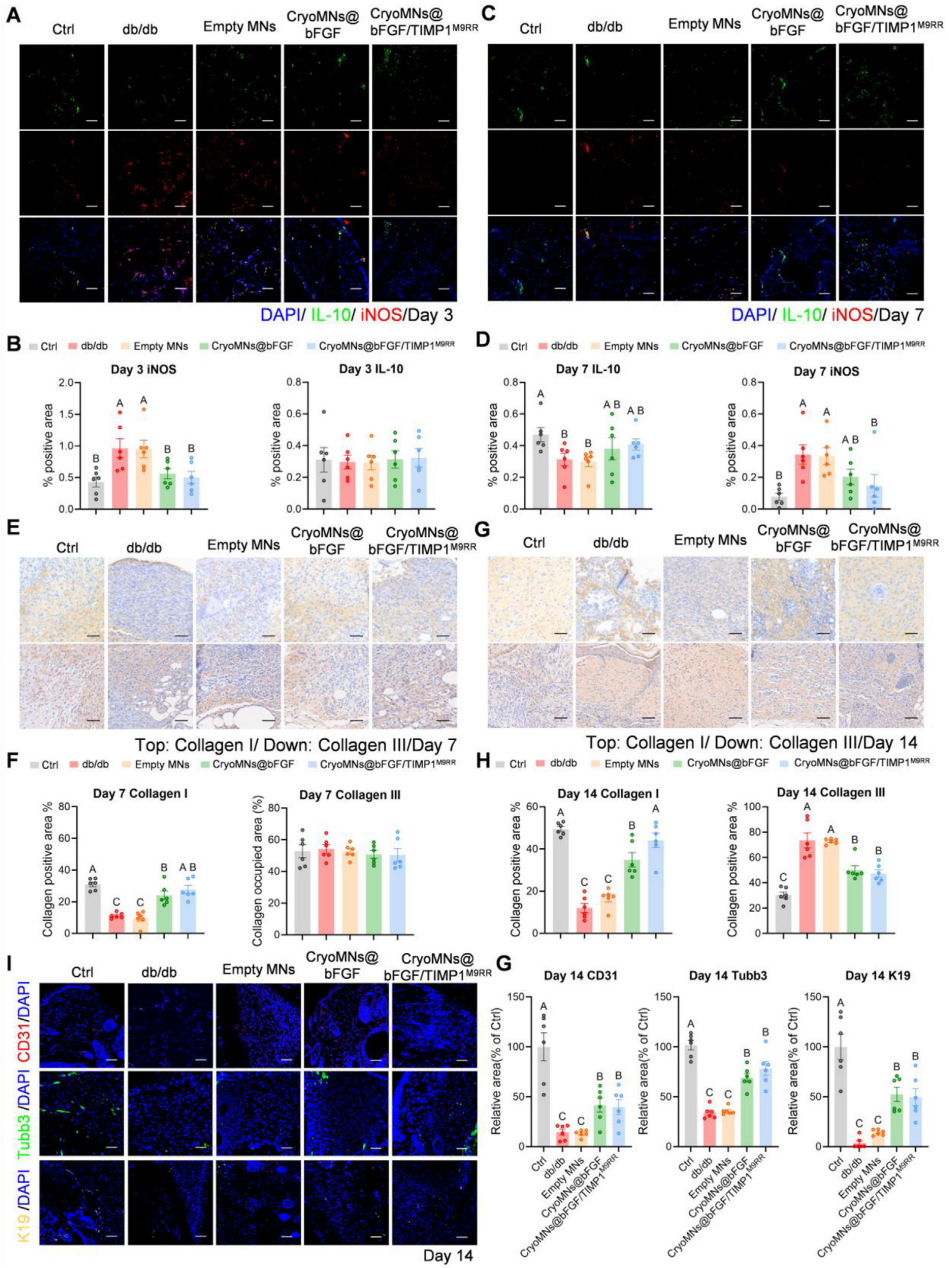
Both TIMP1^M9RR^ and bFGF modulate immune response, collagen remodeling, and tissue regeneration in diabetic wounds. (A) Representative images showing the expression of IL‐10 (green) and iNOS (red) in wound regions from mice in different groups on day 3. Nuclei are stained with Hoechst 33342 (blue). Scale bars: 50 µm. (B) Quantitative analysis of the IL‐10 and iNOS positive area percentage on day 3. n = 6. (C) Representative immunohistochemical images showing the expression of IL‐10 (green) and iNOS (red) in wound regions from mice in different groups on day 7. Nuclei are stained with Hoechst 33342 (blue). Scale bars: 50 µm. (D) Quantitative analysis of the IL‐10 and iNOS positive area percentage on day 7. n = 6. (E) Representative images showing the expression of collagen I and collagen III in the wound region from mice in different groups on day 7. Scale bars: 100 µm. (F) Quantitative analysis of collagen I and collagen III positive area percentage on day 7. n = 6. (G) Representative images showing the expression of collagen I and collagen III in the wound region from mice in different groups on day 14. Scale bars: 100 µm. (H) Quantitative analysis of the collagen I and collagen III positive area percentage on day 14. n = 6. (I) Representative immunofluorescent staining of CD31 (red), β3‐Tubulin (TUBB3, green), and Cytokeratin 18 (K19, yellow) in the wound regions of mice in different groups on day 14. Nuclei are visualized with Hoechst 33342 (blue). Scale bars: 50 µm. (J) Quantitative analysis of CD31, TUBB3, and K19 positive area percentage on day 14. n = 6. Data are shown as mean ± s.e.m.; One‐way ANOVA with Fisher's LSD test (E, J, I); Values sharing the same letter are not significantly different (p > 0.05).

Quantitative analysis of the iNOS‐positive area revealed that both cryoMNs treatment groups had a significantly lower proportion than the db/db and empty cryoMNs groups on day 3 (Figure 8A,B). Notably, this significant reduction persisted until day 7 in the CryoMNs@bFGF/ TIMP1^M9RR^ group (Figure 8C,D). In addition, the percentage of IL‐10‐positive area on day 7 was significantly decreased in the db/db and empty cryoMNs groups. In contrast, levels in both cryoMNs therapeutic groups were similar to those in the control group (Figure 8A–D). In addition, immunofluorescence staining of the M2 marker CD206 and the M1 marker CD86 showed elevated M1 polarization in db/db and empty cryoMNs wounds on day 7. Both therapeutic cryoMNs formulations decreased this elevation (Figure S8C,D).

Collagen subtype analysis showed that on days 7 and 14, the Col I‐expression area in both cryoMN therapeutic groups was significantly higher than that in the db/db and empty cryoMN groups (Figure 8E–H). However, only the CryoMNs@bFGF/ TIMP1^M9RR^ group achieved a Col I‐positive area proportion comparable to that of the WT group on day 14 (Figure 8G,H). For the proportion of Col III‐positive area, no significant difference was observed among groups on day 7 (Figure 8E,F). On day 14, the Col III proportion in the db/db and empty cryoMNs groups was significantly higher than that in the control group (Figure 8G,H). We further detected the myofibroblast marker α‐SMA on day 14 via IHC. α‐SMA signal intensity was markedly reduced in the db/db and empty cryoMNs groups, while both treatment groups restored α‐SMA expression to near control levels (Figure S6E,F).

Furthermore, the percentages of CD31‐, TUBB3‐, and K19‐positive areas in the CryoMNs@bFGF and CryoMNs@bFGF/ TIMP1^M9RR^ groups were significantly higher than those in the db/db and empty MNs groups on day 14 (Figure 8I,G).

Collectively, both cryoMNs formulations markedly accelerate diabetic wound regeneration. Co‐administration of TIMP1^M9RR^ exerts no suppressive influence on bFGF's therapeutic effects, as none of the wound healing biomarkers displayed significant attenuation.

### 2.7 Biological Safety Evaluation

To assess the biosafety of LNP‐ and CryoMNs‐based formulations, histopathological analysis of major organs showed no obvious abnormalities in treated mice compared with the db/db model group (Figure S7A). Serum biochemical assays including liver function markers (AST, ALT, TBIL, DBIL) and renal function markers (CREA, UREA) revealed no statistically significant differences between LNP‐treated groups and the db/db group. CryoMNs‐based formulations produced similarly benign profiles, with only a minor AST fluctuation in the empty CryoMNs group (Figure S7B). Collectively, both LNP and CryoMNs delivery systems exhibit favorable biosafety profiles, confirming their suitability for diabetic wound‐healing applications.

## 3 Discussion

This study introduces M9RR, a protein‐release system in which engineered cells serve as depots for the on‐demand release of therapeutic proteins. Upon in situ delivery of the encoding sequence, engineered cells release the therapeutic protein specifically in response to elevated MMP‐9 activity within the pathological microenvironment. The efficacy of this MMP‐9‐responsive strategy was demonstrated using both LNP‐based delivery and CryoMNs‐based co‐delivery with bFGF, resulting in significantly enhanced wound healing in diabetic models.

Our rationale for targeting MMP‐9 in this study is grounded in extensive clinical and molecular evidence. Over decades, clinical studies have consistently demonstrated that MMP‐9 levels are significantly elevated in diabetic wounds, while the expression of its primary endogenous inhibitor, TIMP1, is markedly reduced [5, 31, 32]. This disrupted MMP‐9/TIMP1 balance has been closely associated with delayed wound healing [5]. In this study, we confirmed this imbalance through immunohistochemistry in db/db mice and further validated it by reanalyzing publicly available single‐cell RNA sequencing data from foot ulcer samples. The scRNA‐seq analysis revealed that M1 macrophages are the main cellular source of MMP‐9 expression, and that M1 macrophages from non‐healing DFUs express the highest levels of MMP‐9 compared to those from healthy donors and other diabetic patient samples. Meanwhile, M1 macrophages from non‐healing DFUs exhibited markedly lower levels of TIMP1 compared to those from healing DFUs, suggesting that elevation of TIMP1 may contribute to the healing phenotype. This pattern of TIMP1 suppression in non‐healing wounds appears to be part of a broader inflammatory dysregulation, as similar suppression has also been reported in macrophages from conditions such as ankylosing spondylitis [33].

The expression of MMP‐9 is dynamic throughout the wound healing process [34]. We observed that in normal wounds, MMP‐9 levels peaked on day 3 and decreased by day 14. In contrast, diabetic wounds showed no significant early change, but MMP‐9 levels rose markedly by day 7 and remained high through day 14. These two distinct temporal profiles of MMP‐9 expression in healthy and diabetic wound samples are consistent with established findings in the literature [35]. Importantly, the dynamic changes in MMP‐9 expression in diabetic wounds highlight the critical need for therapeutic strategies that respond in real time to fluctuations in MMP‐9 levels, thereby enabling adaptive drug release.

To achieve real‐time responsiveness, a key strategy is to exploit the well‐characterized proteolytic activity of MMP‐9. Its robust ability to cleave specific peptide sequences has been extensively documented [36]. Building on this mechanism, numerous MMP‐9‐responsive biomaterials have been developed by incorporating MMP‐9‐cleavable peptides or substrates (e.g., gelatin), demonstrating therapeutic potential for diseases with MMP‐9‐dysregulation diseases [37, 38, 39, 40].

In contrast to these material‐centric approaches, our strategy is inspired by recent advances in synthetic biology that enable the programming of cellular behavior. By leveraging a deeper understanding of intracellular protein trafficking, researchers have begun to engineer molecular‐level response systems by co‐opting natural signaling pathways. For example, the RUSH system controls protein localization through engineered secretory and retention signals, demonstrating the feasibility of using the cell membrane as a controllable protein depot [18]. Building on this concept, we designed M9RR as a cell‐based delivery platform that combines the precision of MMP‐9‐responsive elements with the autonomous, dynamic nature of engineered cells, thus moving beyond static drug carriers toward intelligent therapeutic factories. In vitro M9RR characterization validated this design. The system performed as intended, with target protein release being dependent on the presence of active MMP‐9. Furthermore, M9RR demonstrated substrate specificity, broad compatibility, and reversibility, establishing a critical foundation for its potential in vivo application.

Although in vitro experiments validated the universal functionality of the M9RR system in NIH3T3, HaCaT, HEK293T, COS1, and HeLa cells, prominent quantitative differences in MMP‐9‐stimulated SEAP secretion were detected across these cell lines (Figure 2F). Cell‐type‐dependent transfection and fusion protein overexpression efficiency are central sources of such variability. Transfection efficiency of plasmids or lipid carriers is inherently biased by cell lineage, and previous research has confirmed that vector type and cDNA origin are strongly correlated with transgene induction potency [41, 42]. Furthermore, cell‐specific disparities in transcriptional regulation, cytoplasmic mRNA turnover, and translational competence limit the total quantity of membrane‐anchored M9RR substrates accessible to extracellular MMP‐9, ultimately leading to cell‐type‐specific disparities in the system's response efficiency.

To translate this system into an in vivo therapy, we employed LNPs, a clinically validated mRNA delivery platform known for its high efficacy and favorable safety profile [43]. In vivo small animal imaging showed that LNP‐mediated transgene expression persisted for approximately 7 days. Although the M9RR system facilitated multidimensional tissue repair within this window, the prolonged healing process of diabetic wounds suggests that a longer therapeutic duration may be necessary for optimal outcomes. Therefore, future studies should explore the use of viral vectors, such as lentivirus or adeno‐associated virus (AAV), to achieve sustained transgene expression, thereby providing continuous support throughout the entire repair process.

After intervention of diabetic wounds via cryomicroneedle co‐delivery of bFGF and TIMP1^M9RR^, the wound closure rate and collagen deposition on day 14 were superior to those in the single bFGF monotherapy group, implying that combined treatment may confer modest regenerative benefits. Nevertheless, in vitro experiments using NIH3T3 fibroblasts, co‐administration of TIMP1 and bFGF failed to further boost phosphorylation signals of core downstream mediators of the bFGF cascade (e.g., AKT and ERK) (data not shown). This discrepancy between in vitro molecular profiles and in vivo tissue phenotypes likely arises from the limitations of 2D single‐cell cultures. They lack keratinocyte‐fibroblast‐macrophage crosstalk and fail to recapitulate the diabetic wound microenvironment with dynamic MMP‐9 turnover and ongoing ECM turnover. Future studies may construct 3D skin organoids to interrogate TIMP1/bFGF regulatory interactions under high‐MMP‐9 wound conditions.

Notably, the free bFGF plus free TIMP1 control group was not included in our in vivo experiments, primarily because the total amount of TIMP1 produced by the TIMP1 system cannot be accurately quantified in vivo. CryoMNs‐mediated mRNA transfection induces endogenous TIMP1 synthesis in a dynamic, MMP‐9‐dependent manner, exhibiting release kinetics that are completely distinct from those of direct exogenous delivery of recombinant TIMP1. Accordingly, free TIMP1 treatment fails to provide valid and comparable control results due to unmatched drug profiles and unavoidable confounding variables. Future work will establish elaborate control groups to distinguish the independent contributions of the bFGF/TIMP1 combination, cryomicroneedle delivery, and the M9RR responsive module.

Finally, the MMP‐9‐responsive protein release system described in this study is not limited to DFU treatment, but represents a versatile platform with broad applicability. By selecting specific cleavage sequences for different MMP family members and substituting targeted therapeutic proteins, this system can be adapted to various pathological microenvironments characterized by elevated MMP expression, thereby meeting the requirements for targeted protein delivery. This technical approach is anticipated to enable local, precise treatment of multiple diseases, including but not limited to cardiovascular diseases [44], tumor invasion and metastasis [45], and chronic inflammatory diseases [46], offering a therapeutic paradigm for conditions that require precise microenvironmental regulation.

## 4 Conclusion

This study developed an MMP‐9‐responsive protein release system that enables on‐demand TIMP1 release via proteolytic cleavage, thereby addressing the MMP‐9/TIMP1 imbalance in DFUs. M9RR exhibited MMP‐9 specificity, broad applicability across mammalian cell types, and reversibility, protecting HaCaT and BJ cells from MMP‐9‐induced damage in vitro. In db/db mice, LNP‐delivered TIMP1^M9RR^ significantly enhanced wound contraction, granulation tissue formation, and collagen remodeling by dynamically neutralizing excessive MMP‐9. Moreover, CryoMNs‐mediated co‐delivery of TIMP1^M9RR^ and bFGF achieved promising healing effects, particularly in regulating inflammation and promoting collagen maturation. Both LNP and CryoMNs formulations showed favorable biosafety. As a modular platform, M9RR can be adapted to other MMP‐related diseases, offering a precise, responsive therapeutic strategy for DFUs and beyond.

## 5 Methods

### 5.1 RNA Extraction, Library Construction, and Sequencing

Total RNA was extracted from in vitro samples (HaCaT and BJ cells, treated with 10 ng/ml MMP9 with or without transfection with 1 ng/ml LNP) and in vivo samples (db/db mice) using TRIzol Reagent. In vitro, cells were seeded at 2×10^5^ cells per well in 6‐well plates and received 24 h of TIMP1^M9RR^ mRNA‐LNP or EGFP mRNA‐LNP treatment (1 ng/mL final mRNA), then incubated with 10 ng/mL MMP‐9 for another 24 h before RNA isolation. Sequencing libraries were prepared and sequenced on the Illumina NovaSeq 6000 platform (Novogene Co., Ltd., China). Data pre‐processing was performed using the NovoMagic online platform (Novogene Co., Ltd., China). Briefly, raw FASTQ data were first subjected to quality assessment and adapter trimming. Clean reads were then aligned to the human (GRCh38) and mouse (GRCm39) reference genomes using HISAT2 (v2.0.5) [47]. Subsequently, transcript abundances were quantified using (v1.5.0‐p3) to estimate gene and isoform expression levels [48]. Raw counts were downloaded from the NovoMagic platform for in‐house differential expression (DE) analysis. DESeq2 (v1.38.3) was used to identify DEGs with the criteria of |log_2_FoldChange| ≥ 0.585 (1.5‐fold) and adjusted p‐value < 0.05 [49]. The identified DEGs were subjected to GO analysis and GSEA using clusterProfiler (v4.7.1) [50]. Raw and processed sequencing data have been deposited in the GEO database (accession number: PRJNA1380755).

### 5.2 Re‐Analysis of Publicly Available Data Sets

Publicly available bulk RNA‐seq and scRNA‐seq data sets (GSE199939 and GSE165816) were downloaded from GEO databases for re‐analysis [51, 52]. For the GSE199939 dataset, the TPM count matrix was obtained, and the expression of MMP genes in samples from diabetic foot ulcer patients and healthy controls was visualized using heatmaps. The processed scRNA‐seq data from GSE165816 were downloaded and re‐analyzed using Seurat (v4.4.0) for clustering, cell‐type annotation, and gene expression visualization [53].

### 5.3 Plasmid Construction

Plasmids used in this study were constructed via Gibson assembly using the Gibson Assembly Cloning Kit (New England BioLabs, MA, USA). All DNA fragments were generated by standard PCR protocols. All constructs were verified by Sanger sequencing (BGI, Hong Kong). Detailed information on the plasmids is included in Table S1.

### 5.4 Cell Culture and Transfection

The COS1(ATCC, CRL‐1650, RRID: CVCL_0223), NIH/3T3 (Beyotime, C7204, RRID: CVCL_0594), HeLa (Beyotime, C6330, RRID: CVCL_0030), HaCaT (Beyotime, C6282, RRID: CVCL_0038), BJ (ATCC, CRL‐2522, RRID: CVCL_3653), HEK293T (Beyotime, C6008, RRID: CVCL_0063) cells were cultured in high‐glucose Dulbecco's Modified Eagle Medium (DMEM; Gibco, cat. no. 11965118) supplemented with 10% fetal bovine serum (FBS; Gibco, USA). Cells were maintained in a humidified incubator at 37°C with 5% CO_2_ (Thermo Fisher Scientific, USA) and passaged regularly using 0.25% trypsin‐EDTA (Gibco, USA). Cells in the logarithmic growth phase were selected for subsequent experiments. All cell lines were confirmed to be contamination‐free.

For cell transfection, the indicated plasmids were transfected using Lipomaster 2000 (Vazyme, TL201) or by co‐culturing cells with SM102‐based LNP‐encapsulated mRNA (Hemu Biotech, Beijing). DMEM supplemented with different matrix metalloproteinase (MMP) enzymes was prepared by adding activated MMPs at the indicated concentrations to FBS‐free DMEM. MMP2 (MCE, HY‐P73810) and MMP‐9 (MCE, HY‐P73807) were used in this study.

### 5.5 MMP‐9 Activation

A 100 mM stock solution of aminophenylmercuric acetate (APMA) (MCE, HY‐148905) was prepared in DMSO. MMP‐9 (100 µg/mL) was prepared in TCNB buffer (Shanghai Shangbao, T18065.100). The APMA stock solution and MMP‐9 solution were mixed at a 1:100 volume ratio, then incubated at 37°C for 24 h. The activated MMP‐9 protein was used immediately after activation.

### 5.6 Analytical Assays

The expression of the reporter gene SEAP was quantified using the Phosphate‐Light SEAP Reporter Gene Assay System (Invitrogen, cat. no. T1017) according to the manufacturer's instructions. MMP‐9 activity was measured by incubating the activated MMP‐9‐containing solution with the MMP‐9 fluorescent substrate MOCAc‐PLGL(Dpa)ARGLP‐1 (MedChemExpress, HY‐131498) for 6 h. Fluorescence signal intensity was then measured following the manufacturer's instructions. TIMP1 levels in cell supernatants were quantified using the Mouse TIMP1 ELISA Kit (Beyotime, PT8830) following the manufacturer's protocols. To quantify the activity of TIMP1 on neutralization of MMP‐9, the HEK293T cells transfected with or without the TIMP1^M9RR^ plasmid were incubated with different concentrations of activated MMP‐9 for 12 h. Then, MMP‐9 activity in the supernatant was quantified using the fluorescent MMP‐9 substrate MOCAc‐PLGL(Dpa)ARGLP‐1.

### 5.7 Cell Immunofluorescence Staining and Imaging

To examine the subcellular localization of the fusion protein, coverslips (174950, Thermo Fisher Scientific) were placed in 12‐well plates, and cells were seeded on the coverslips at a ratio of 1:5. After 24 h, cells were transfected with the indicated plasmids using Lipomaster 2000 (Vazyme, TL201).

At 24 h post‐transfection, cells were stained with Hoechst 33342 (1:1000, Beyotime, C1022) for 10 min to label nuclei, and excess dye was removed by washing with phosphate‐buffered saline (PBS). Cell membranes and nuclei were labeled using CellMask Membrane Stain (Invitrogen, C10045) and Hoechst 33342 (Beyotime, C1022), respectively, according to the manufacturers’ instructions. Imaging was performed using an Olympus FV1200 Inverted Confocal Microscope with excitation wavelengths of 488 nm for enhanced green fluorescent protein (EGFP), 405 nm for Hoechst 33342, and 555 nm for CellMask.

For MMP‐9‐triggered EGFP release from the cell membrane, HEK293T cells were seeded on the coverslips at a ratio of 1:5. On the second day, the cells were transfected with the EGFP‐P2 plasmid. At 24 h post‐transfection, cells were treated with 1000 ng/mL activated MMP‐9 for 6 h, and imaging was performed. Three independent fields of view were selected for quantification of fluorescence intensities of EGFP and CellMask using ImageJ software. The relative EGFP fluorescence intensity was calculated as the ratio of EGFP signal to CellMask fluorescence intensity, followed by statistical analysis.

### 5.8 MMP‐9 Responsiveness Assay

Cells were seeded in 24‐well plates at a ratio of 1:4. After 24 h, cells were transfected with the indicated plasmids. 24 h after transfection, the cells were starved overnight in serum‐free medium. Subsequently, the cells were treated with specified concentrations of MMP9 or MMP2 and 50 µmol/L ZnCl_2_. Cell supernatants were collected at 0, 1, 2, and 6 h to measure SEAP or TIMP1 activity.

For the periodic MMP9 treatment experiment, the transfected cells were alternately treated with serum‐free medium containing MMP9 plus 50 µmol/L ZnCl_2_ and serum‐free medium alone, and samples were collected at the designated time points.

### 5.9 Detection of LNP Efficiency

HaCaT and BJ cells were seeded at a density of 5 × 10^4^ cells per well in 24‐well plates. At about 70% confluency, cells were transfected with EGFP mRNA‐loaded liposomes (final mRNA concentration: 1 ng/mL). EGFP expression was observed using a fluorescence microscope (IX73, Olympus, Japan) on day 1, 3, and 7 post‐transfection.

### 5.10 Quantitative Polymerase Chain Reaction (qPCR) Assay

HaCaT and BJ cells were seeded in 6‐well plates at a density of 2×10^5^ cells per well. When cell confluency reached 80%, cells were divided into groups for treatment: control group (no treatment), Empty LNP group, and TIMP1^M9RR^ group (treated with LNPs ‐TIMP1^M9RR^ mRNA). Each treatment group was further subdivided into subgroups co‐treated with 10 or 100 ng/mL activated MMP‐9. At 48 h post‐treatment, qPCR was performed to detect the expression of target genes. Primer sequences are listed in Table S2.

### 5.11 EdU Proliferation Assay

HEK293, HaCaT, or BJ cells were seeded in 24‐well plates. Cells were transfected at 70% confluency with the plasmids as indicated. At 24 h post‐transfection, cells were treated with activated MMP‐9 (1000 ng/mL for HEK293 cells; 100 ng/mL for HaCaT and BJ cells) for 24 h. Cell proliferation was assessed using the BeyoClick EdU‐555 Cell Proliferation Assay Kit (Beyotime, C0075S) or the Click‐iT EdU Alexa Fluor 594 Imaging Kit (Thermo Fisher Scientific, USA) according to the manufacturer's instructions. Cells were observed and imaged under a fluorescence microscope, and three independent fields of view were selected. The number of EdU‐positive cells and total cells was counted using ImageJ software, and the EdU‐positive rate was calculated as (number of EdU‐positive cells/total number of cells) × 100%.

### 5.12 Flow Cytometry Analysis

Cells after Edu labeling were collected by trypsinization and fixed with 4% paraformaldehyde (PFA). The proportion of EdU‐positive cells was measured using a BD FACSymphony A5.2 SORP Cell Analyzer. Experiments were independently repeated three times.

### 5.13 Cell Scratch Assay

HaCaT and BJ cells were seeded in 6‐well plates. At 24 h post‐seeding, cells were transfected with the specified plasmids and cultured until forming a confluent monolayer. A uniform scratch was created in the cell monolayer using a sterile 200 µL pipette tip. Cells were gently washed twice with PBS to remove detached cells, and then cultured in serum‐free DMEM supplemented with 100 ng/mL activated MMP‐9. At 0‐, 12‐, and 24‐h post‐scratching, cells were fixed with 4% PFA, stained with crystal violet (Beoytime, C0121), and imaged under an inverted microscope (IX73, Olympus, Japan). The area of the scratched region was measured using ImageJ software, and the scratch wound coverage was calculated as (scratch area at a specific time point/scratch area at 0 h) × 100%. Each sample was set up in three technical replicates, and experiments were independently repeated three times.

### 5.14 Preparation of CryoMNs

Hyaluronic acid (molecular weight: 200 000 Da; Yeasen, 61009ES08) was dissolved in deionized water at a concentration of 50 mg/mL to serve as the matrix material for CryoMNs. mRNA‐loaded LNPs (2 µg mRNA per microneedle) and/or Recombinant Human bFGF (Yeasen, 91334ES50; 1 µg per microneedle) were added to the hyaluronic acid solution, and the mixture was thoroughly homogenized to prepare the pre‐freezing solution for microneedles. The pre‐freezing solution was injected into polydimethylsiloxane (PDMS) microneedle molds (needle height: 650 µm; needle base diameter: 320 µm; needle spacing: 500 µm) and pre‐frozen at −80°C for 2 h. CryoMNs loaded with mRNA‐loaded LNPs were obtained by demolding. Blank CryoMNs without LNPs were prepared as controls.

Mechanical properties of CryoMNs were tested using a universal testing machine (Instron 5944, Instron, USA). Force‐displacement curves were recorded.

### 5.15 Detection of Transfection Efficiency of LNPs‐EGFP mRNA‐Loaded CryoMNs

HaCaT and BJ cell lines were treated with LNP‐EGFP mRNA or LNP‐EGFP mRNA‐loaded CryoMNs (final mRNA concentration: 1 ng/mL). At 24 h post‐treatment, cells were stained with Hoechst 33342 to label nuclei, and EGFP expression in skin tissues was observed under an Olympus FV1200 Inverted Confocal Microscope. Three independent view fields were selected, and the proportion of EGFP‐positive cells was quantified using ImageJ software, which was defined as the in vitro transfection efficiency.

### 5.16 Penetration Test of CryoMNs

Prepared CryoMNs were applied to freshly excised mouse skin. After the microneedles melted, the skin tissues were fixed, embedded, sectioned (thickness: 5 µm), and stained with hematoxylin and eosin (H&E). Penetration traces of the microneedles in the skin were observed. In addition, agarose hydrogel was used to simulate human skin tissue for penetration experiments to evaluate the efficiency of CryoMNs in delivering protein factors and nanoliposomes. Briefly, 1% agarose gel (Yeasen, 10208ES60) was prepared at room temperature. CryoMNs containing 0.1% rhodamine B (Mackin, R817226) were pressed vertically onto the gel surface. Penetration of the microneedles into the gel was observed and imaged at 0, 30, 60, 120, 180, and 240 s. The stained area of rhodamine B in the cross‐section was quantified using ImageJ software.

### 5.17 Animal Experiments

All animal experiments were approved by the Ethics Committee of The Chinese University of Hong Kong (approval number: 21‐212‐MIS) and licensed by the Department of Health, Hong Kong.

To establish a diabetic mouse wound healing model, 8‐week‐old db/db diabetic mice or C57BL/6J mice (20–25 g) were acclimatized for 1 week before model establishment. Mice were anesthetized with isoflurane, and their dorsal fur was shaved and disinfected. Full‐thickness skin defects (2 wounds per mouse) were created on both sides of the dorsal midline using a sterile 6 mm biopsy punch. After hemostasis, mice were randomly divided into groups for different treatments (3 mice per group, 6 wounds total). For the C57BL/6J control group and untreated group, wounds were coated with normal saline. For the TIMP1 group, wound beds were directly injected with 2 µg LNPs‐TIMP1 mRNA. For the TIMP1^M9RR^ group, wound beds were directly injected with 2 µg LNP‐TIMP1^M9RR^ mRNA. For the empty CryoMNs group, wounds were treated with blank CryoMNs. For the CryoMNs@bFGF group, wounds were treated with 1 µg bFGF‐loaded CryoMNs. For the CryoMNs@bFGF/TIMP1^M9RR^ group, wounds were treated with CryoMNs loaded with bFGF and LNP‐TIMP1^M9RR^ mRNA. CryoMNs were applied by vertically pressing onto the wound to ensure complete penetration into the epidermis.

Wound appearance was photographed on day 0, 3, 7, and 14 day post‐operation, and wound area was measured using ImageJ software. Mice were euthanized on day  3, 7, and 14 post‐operation, and skin tissues (including wounds and the surrounding 1 cm area) were collected for subsequent histological analysis and sequencing. Wound tissues of identical dimensions were minced, digested, and centrifuged, and the resulting supernatants were collected for ELISA analysis. All operations strictly followed the manufacturer's protocols (Cat. No. PT883, PM733; Beyotime Biotechnology).

### 5.18 In Vivo Imaging

db/db mice were divided into four groups: a PBS control group (local subcutaneous injection of PBS), an LNPs‐Luciferase mRNA group (local subcutaneous injection of 2 µg luciferase mRNA encapsulated in LNPs), an LNP‐Luciferase mRNA‐loaded CryoMNs group, and a blank CryoMNs control group. After dorsal fur removal and disinfection, mice were subjected to subcutaneous injection or CryoMNs application. On 1‐, 3‐, and 7‐day post‐treatment, D‐luciferin (150 mg/kg, PerkinElmer, USA) was intraperitoneally injected. 10 min later, fluorescence signal intensity in the dorsal wound area of mice was measured using an in vivo small animal fluorescence imaging system (IVIS Spectrum, PerkinElmer, USA). Luciferase signal intensity was quantified to evaluate the in vivo delivery efficiency and expression persistence of LNP‐mRNA via wound bed injection and CryoMNs.

### 5.19 Histopathological Analysis

On 3‐, 7‐, and 14‐day post‐treatment, skin tissues encompassing the wound and the surrounding 1 cm area were collected. Tissues were fixed in 4% paraformaldehyde (PFA) for 24 h. After fixation, samples were embedded in paraffin and sectioned with 5 µm thickness. Sections were stained with hematoxylin and eosin (H&E), Masson's trichrome, periodic acid‐Schiff (PAS), or Sirius red. For H&E staining, the morphology of the wound tissue was observed, including epidermal regeneration and granulation tissue formation, etc. The wound width and tissue thickness (in the center area of the wound) were quantitatively analyzed using ImageJ software. For Masson staining, the percentage of collagen area (collagen area/total field area × 100%) was calculated using ImageJ software. For Sirius Red staining, collagen fibers were observed under polarized light. Type I collagen appeared red or yellow, and type III collagen appeared green. The orientation of collagen fibers (the angle between the fiber and the long axis of the wound) was analyzed using OrientationJ software [54]. The ratio of Col I to Col III was quantitatively analyzed using ImageJ. All images were captured by a ZEISS Axioscan Microscope Slide Scanner.

Paraffin sections were subjected to IHC staining as previously reported and observed using a ZEISS Axioscan 7 Microscope Slide Scanner [55]. The primary antibodies used were: TIMP1 antibody (1:200, ABclonal, A1389), MMP‐9 antibody (1:200, ABclonal, A25299), collagen I antibody (1:200, ABclonal, A24112), and collagen III antibody (1:200, ABclonal, A3795). The percentage of positive area (stained brownish‐yellow) was quantified using ImageJ software.

Paraffin sections were subjected to IF staining as described [56]. After antigen retrieval of paraffin sections, primary antibodies against IL10 (1:200, Santa Cruz, sc‐365858), iNOS (1:200, CST, 13120T), CD31 (10 ug/mL, R&D systems, AF3628), TUBB3 (1:100, CST, 5568T), and K19 (1:200, Santa Cruz, sc‐376126) were added, and sections were incubated at 4°C overnight. Sections were then incubated with Alexa Fluor 488‐ (1:1000, Invitrogen, A32723) or Alexa Fluor 554‐conjugated secondary antibodies (1:1000, Invitrogen, A32732) at room temperature for 1 h in the dark. Nuclei were stained with DAPI (D9542, Sigma). Images were captured using an Olympus FV1200 Inverted Confocal Microscope with a SIM scanner, and the percentage of positive area for the target protein was quantified using ImageJ software.

### 5.20 Safety Evaluation

At the end of the in vivo experiment, serum samples were collected from mice in each group. Liver and kidney function indicators, including alanine transaminase (ALT), aspartate transaminase (AST), total bilirubin (TBIL), direct bilirubin (DBIL), urea, and creatinine (CREA), were detected using an automatic biochemical analyzer (Cobas 8000, Roche, Switzerland). After euthanasia, tissues including heart, liver, spleen, lung, and kidney were collected, stained with H&E, and observed for histological changes under a ZEISS Axioscan 7 Microscope Slide Scanner.

### 5.21 Statistical Analysis

All experimental data were statistically analyzed and graphically presented using GraphPad Prism 9.0 software (GraphPad Software, USA). Quantitative data were expressed as mean ± standard error of the mean (mean ± s.e.m). For data normalization, values from each experiment were expressed as fold‐change relative to the control group (set to 1) or to the baseline time point (0 h, set to 1) as indicated in the figure legends. For bioluminescence imaging data, the average radiance values (p/s/cm^2^/sr) were log_10_‐transformed prior to statistical analysis. Comparisons between two groups were performed using the unpaired t‐test; one‐way analysis of variance (ANOVA) followed by Fisher's LSD test or two‐way ANOVA followed by Fisher's LSD test was used for comparisons among multiple groups. A *p*‐value < 0.05 was considered statistically significant, and a *p*‐value < 0.01 was considered extremely statistically significant. All experiments were independently repeated at least three times to ensure the reliability of the results.

## Author Contributions

Supervision: H.Z., Conceptualization: H.Z., X.L., and Y.Z., Investigation: Y.Z., J.N.D., M.Y.C., T.T.F., X.F.Q., D.Q.C., J.M.S., G.L., P.S., W.Y.C., and X.L., Writing – original draft: Y.Z., X.L., M.Y.C, and J.N.D., Writing – review and editing: H.Z., X.L., Y.Z., and M.Y.C.

## Conflicts of Interest

The authors declare no conflicts of interest.

## Supporting information




**Supporting File 1**: advs77810‐sup‐0001‐SuppMat.docx.

**Supporting File 2**: advs77810‐sup‐0002‐Tables.zip.

## Data Availability

The authors have nothing to report.
