# Supplementary material for: In Situ Genetic Engineering Facilitates MMP‐9‐Responsive TIMP1 Release Through Proteolytic Cleavage to Boost Diabetic Wound Healing
Source: Adv Sci (Weinh). 2026 Sep 27:e24328. Online ahead of print. doi: 10.1002/advs.202524328 (PMC13616301; doi:10.1002/advs.202524328)
Supplement: Supplementary file 1 [file ADVS-9999-e24328-s001.docx]

Supporting Information

**In Situ Genetic Engineering Facilitates MMP-9-Responsive TIMP1 Release through Proteolytic Cleavage to Boost Diabetic Wound Healing**

*Yuan Zhang^ac#^, Jiani Deng^a#^, Man Yee Cheung ^a^, Tingting Fu^d^, Xufeng Qi^d^, Dongqing Cai^d^, Jianmin Sun^e^, Gang Lu^a,g,h^, Peng Shi^f,g^, Wai-Yee Chan^a,g,h^, Xu Li**^b^*, Hui Zhao^a,g,h^**

^a^ Key Laboratory for Regenerative Medicine of the Ministry of Education of China, School of Biomedical Sciences, Faculty of Medicine, The Chinese University of Hong Kong, Shatin, Hong Kong SAR, People's Republic of China.

^b^ Beijing Jishuitan Hospital, Capital Medical University, Beijing, China.

^c^ National Infrastructures for Translational Medicine (PUMCH), Institute of Clinical Medicine, Peking Union Medical College Hospital, Chinese Academy of Medical Sciences & Peking Union Medical College, Beijing, 100730, China.

^d^ Key Laboratory of Regenerative Medicine of Ministry of Education, Department of Developmental & Regenerative Biology, Jinan University, Guangzhou, Guangdong 510632, China.

^e^ Department of Pathogen Biology and Immunology, School of Basic Medical Sciences, Ningxia Medical University, No. 1160 Shengli Street, Yinchuan 750004, China.

^f^ State Key Laboratory of Genetic Resources and Evolution/Yunnan Key Laboratory of Biodiversity Information, Kunming Institute of Zoology, The Chinese Academy of Sciences, Kunming, China.

^g^ Kunming Institute of Zoology, Chinese Academy of Sciences, The Chinese University of Hong Kong Joint Laboratory of Bioresources and Molecular Research of Common Diseases, Hong Kong SAR, China.

^h^ Hong Kong Branch of CAS Center for Excellence in Animal Evolution and Genetics, The Chinese University of Hong Kong, New Territories, Hong Kong SAR, China.

\# Yuan Zhang and Jiani Deng contributed equally to this work.

* Corresponding authors: Prof. Hui Zhao, zhaohui@cuhk.edu.hk; Dr. Xu Li, [lixu0610@126.com](mailto:lixu0610@126.com)

**Figure S1. (A)** Dotplot showing the marker genes used for cell type annotation. **(B)** UMAPs showing the expression of all detected MMP genes in scRNA-Seq data from foot skin and foot ulcer samples. Data were retrieved from GSE 165816.

**Figure S2. (A)** Measurement of enzyme activity of APMA-treated MMP9 using fluorescent substrate MOCAc-PLGL(Dpa)AR. n=3. **(B)** CCK8 assay showing the time-dependent changes in cell proliferation capacity (HEK293T) after transfection with the TIMP1^M9RR^. **(C)** Representative images of the EdU incorporation assay for cell proliferation (HEK293T) after TIMP1^M9RR^ transfection. **(D)** Statistical results of cell proliferation rate measured by EdU flow cytometry after TIMP1^M9RR^ transfection. Data are presented as mean ± s.e.m.; One-way ANOVA with Fisher’s LSD test (A); Unpaired t-test (E), **P* < 0.05, ***P* < 0.01, and ****P* < 0.001.

**Figure S3. (A-B)** Representative fluorescence images of EGFP in HaCaT (A) and BJ (B) cells transfected with lipid nanoparticles-EGFP mRNA (LNPs-EGFP). Scale bars: 50 µm. **(C-D)** PCA analysis of the expression data of HaCaT samples (C) and BJ samples (D).

**Figure S4. (A)** *In vivo* imaging of C57BL/6 mice at 24 hours without and with application of SM102LNP-luciferase mRNA. **(B)** Comparison of average radiance [p/s/cm2/sr] between two different groups as indicated. n=4. **(C)** Representative immunohistochemical images showing the expression of TIMP1 in wound regions from mice in different groups on day 3 and day 7. Scale bars: 100 µm. **(D)** Quantitative analysis of the TIMP1-positive area percentage on day 3 and day 7. n=6. **(E, F)** ELISA assays of MMP-9 in diabetic wounds. n=4. Data are shown as mean ± s.e.m.; Multiple unpaired t-tests (B); two-way ANOVA with Fisher’s LSD test (D, E, F); values sharing the same letter are not significantly different (p > 0.05).

**Figure S5. (A)** PCA analysis of the expression data from db/db and treatment groups. **(B)** Cross-group analysis showing the overlap of DEGs among HaCaT cells (green), BJ cells (blue), and db/db mouse samples (red). **(C)** Dotplots showing the log2FC of the 49 genes overlapping between human (HaCaT+BJ) and mouse (db/db mice) samples. **(D)** GO term (biological process, BP) enrichment analysis on the overlapped DEGs. **(E)** Representative immunohistochemical images showing the expression of IL6 in the wound region from mice in different groups on day 14. Scale bars: 50 µm**. (F)** Quantitative analysis of the average optical density (AOD) of IL6 on day 3, day 7, and day 14. n=6. **(G)** Representative images showing the expression of CD206 or CD86 (red) in wound regions from mice in different groups on day 3 and day 7. Nuclei are stained with Hoechst 33342 (blue). Scale bars: 50 µm. **(H)** Quantitative analysis of CD206- and CD86-positive area percentage on day 3 and day 7. n=6. **(I)** Representative immunohistochemical images showing the expression of α-SMA in the wound region from mice in different groups on day 14. Scale bars: 50 µm. **(J)** Quantitative analysis of the AOD of α-SMA on day 3, day 7, and day 14. n=6. Data are shown as mean ± s.e.m.; One-way ANOVA with Fisher’s LSD test (H, J); Two-way ANOVA with Fisher’s LSD test (F). P < 0.05, **P < 0.01, and ***P < 0.001. Values sharing the same letter are not significantly different (p > 0.05) (H).

**Figure S6. (A)** Representative immunohistochemical images showing the expression of TIMP1 in wound regions from mice in different groups as indicated on day 3. Scale bars: 100 µm. **(B)** Quantitative analysis of the TIMP-1 positive area percentage on day 3. n=6. **(C)** Representative images showing the expression of CD206 or CD86 (red) in wound regions from mice in different groups on day 3 and day 7. Nuclei are stained with Hoechst 33342 (blue). Scale bars: 50 µm. **(D)** Quantitative analysis of the CD206- and CD86-positive area percentage on day 3 and day 7. n=6. **(E)** Representative immunohistochemical images showing the expression of α-SMA in the wound region from mice in different groups on day 14. Scale bars: 50 µm. **(F)** Quantitative analysis of the AOD of α-SMA on day 14. n=6. Data are shown as mean ± s.e.m. One-way ANOVA with Fisher’s LSD test. Values sharing the same letter are not significantly different (p > 0.05).

 **Figure S7 (A)** H&E staining of tissues (i.e., heart, liver, spleen, lung, and kidney) from different groups after 14 days of the testing period. Scale bar, 200 μm. **(B)** Concentration of serum alanine aminotransferase (ALT), aspartate aminotransferase (AST), urea (UREA), creatinine (CREA), total bilirubin (TBIL), and direct bilirubin (DBIL) in different groups after 14 days of the testing period. n = 6. Data are shown as mean ± s.e.m. One-way ANOVA with Fisher’s LSD test. Values sharing the same letter are not significantly different (p > 0.05).
